# Light-activated 3D covalent organic framework membranes with adaptive pores for CO_2_ recognition and separation

**DOI:** 10.1126/sciadv.adw8452

**Published:** 2025-08-06

**Authors:** Congcong Yin, Muning Chen, Ziyin Zhang, Kai Liu, Jinglin Gao, Xin Zhao, Yuping Wu, Yong Wang

**Affiliations:** ^1^ School of Energy and Environment, Southeast University, Nanjing, 211189 Jiangsu, P. R. China.; ^2^ School of Chemical Engineering, Zhengzhou University, Zhengzhou, 450001 Henan, P. R. China.

## Abstract

Creation of membrane channels capable of recognizing diverse molecules is desirable for advancing molecular transport and separation, but it remains difficult to precisely control their conformation and achieve dynamic adaption to specific molecules. Here, we demonstrate fine-tuning the pore environment within 3D covalent organic framework (COF) that can precisely control its pore size and polarity to recognize CO_2_. The pores are integrated with azobenzene units with photo-adaptable trans-to-cis isomerization, enabling dynamic pore size regulation at angstrom scale. Moreover, the isomerization induces drastic changes in intrapore polarity, which exert profound effects on the intermolecular affinity through the dipole-quadrupole interaction. Featuring these dynamic natures, the 3D COF membrane cooperating the size exclusion effect with a molecular recognition ability toward CO_2_. As a result, light-gating separation experiments show the superior CO_2_ capture performance with a N_2_/CO_2_ selectivity of 27.6. Theoretical calculations reveal that cis-state azobenzene can reduce the electron transfer barrier from azobenzene to CO_2_, thereby providing a molecular recognition pathway.

## Teaser

A molecular recognition pathway is created within the 3D COF membrane, offering adaptive channels for fast CO_2_ separation.

## INTRODUCTION

Covalent organic frameworks (COFs) are porous and crystalline materials that are formed by connecting organic building blocks into extended networks via reticular chemistry (*1*–*3*). Well-defined channels and modifiable pore environment give COFs great chance in offering precise control over structures and functions at molecular level (*4*–*6*). Featuring these advantages, COFs have emerged as vital building blocks for advanced membranes. By manipulating the size and functionality of nanochannels, COFs exhibit distinct properties in molecular separation including solvent purification and gas separation (*7*–*9*). Besides, these frameworks are joined by strong covalent bonds, forming rigid scaffolds and ensuring their operation stability (*10*, *11*). However, it remains a great challenge for COF membranes to distinguish molecular mixtures with close kinetic diameters and physical properties because of the difficulty in constructing channels with ultrahigh precision and expected host-guest interaction. This necessitates structural design that involves fine-tuned micropores and specific affinity toward target molecules.

The dynamic control of the COF conformation is expected to be advantageous if the pore aperture and internal surface chemistry could be precisely tailored under external actions. That structure would be accessed through the introduction of motive groups, which allows the strict and continuous adjustment of pore size and specific interactions with molecules (*12*–*14*). Typically, stimulation with light allows fine spatial and temporal precision than other stimuli and has the advantages of remote controllability and environmental friendliness. As a widely studied light-responsive molecule, azobenzene undergoes isomerization between the extended trans-conformation and bended cis-conformation under the exposure to external lights (*15*–*17*). This triggers precise adjustment of the molecular length from 9.0 to 5.5 Å that required for the control of pore size at angstrom scale (*18*, *19*). Simultaneously, the C─N═N─C dihedral angle is changed from 180° to 95°, accompanied by the enhanced dipole moment to 3 D upon ultraviolet (UV) light exposure, which could strengthen the polar interaction between pores and guest molecules (*20*–*22*). Owing to these structural dynamics, azobenzene and its derivatives have applied in molecular transport and separation. Liu and coworkers (*18*) installed azobenzene groups onto covalent organic networks to construct light-responsive membranes. Their pore size could be remotely shifted upon trans-to-cis isomerization, leading to varied permeation in organic solvent nanofiltration (OSN). However, the weakly crystallized network inevitably deteriorates the pore regularity and impairs the separation precision. Consequently, the manipulated mass transport is exemplified in dye purification in solvents rather than gas separation requiring much higher separation precision, and the challenge persists in establishing a holistic approach to precisely build molecular transport channels. In this regard, Wu and coworkers (*23*) explored a series of photoresponsive COFs by incorporating dangling azobenzene groups as intrapore units. The pore size distributions could be precisely tuned through the azobenzene isomerization, leading to enhanced CO_2_/N_2_ selectivity. To create a broad diversity of pore functionalities, Jiang and coworkers (*24*) integrated tetrafluoroazobenzene units onto COF pore walls as photosensitive sites, which affords obvious changes of the pore size and environment. Upon trans-to-cis isomerization, the polarity of tetrafluoroazobenzene units was greatly increased, leading to enhanced dipole-quadrupole interactions of COFs with CO_2_ molecules. On the basis of these great efforts, it can be anticipated that rational design and isomerization of azobenzene side chains would dynamically control COF pore configuration and achieve fast molecular transport. However, previous works on photoresponsive gas separations were all realized by adsorption rather than membrane separation (*23*, *24*).

To take full advantage of the photoresponsivity of azobenzene, it is necessary to anchor azobenzene units into the COF scaffold that has high porosity and ample active sites. With multidimensional periodic backbones, three-dimensional (3D) COFs are highly prized for their unique structures and functions. Their interpenetrated channels, high porosity, and plentiful accessible sites provide 3D COFs the potential to construct structures having both long-range orderliness and local flexibility (*25*–*27*). Moreover, the structural units are entirely linked with strong covalent bonds, which could further ensure the operation stability (*28*, *29*). Through integrating azobenzene groups into the frameworks, 3D COFs can undergo robust and sufficient structural isomerization, which then lead to desirable molecular transport behaviors. However, there are still some obstacles that block the utilization of these systems. First, the structure of azobenzene is generally altered at two states, which is difficult to create defined pore environment and favor molecular transport. Second, it remains elusive to establish the interaction mechanisms to promote molecular transport because it depends on both binding energies to pore channels and orbital contributions of azobenzene.

Taking these challenges in mind, we here design a 3D COF featuring dynamic azobenzene units to enable remote control of pore geometry and polarity in a continuous manner. The 3D framework with interconnected and spatially open channels permits unimpeded structural modulation. Consequently, light activation on the 3D COF induces precise and quantitative tuning of pore sizes at the angstrom scale. Moreover, this drives efficient trans-to-cis isomerization while preserving the crystallinity, ensuring robust molecular recognition. The azobenzene groups are rationally isomerized with favorable dipole moment, contributing to decent electron affinity between C═O of CO_2_ and C═N of azobenzene. Further advancement of the azobenzene isomerization leads to intensive electron attraction from azobenzene to CO_2_, thereby recognizing CO_2_ molecules through the dipole-quadrupole interaction. By establishing the synergistic effect of size-sieving and light-activated molecular recognition, the 3D COF membrane can selectively capture CO_2_ over gases with low polarizabilities.

## RESULTS

### Light-activated structural transformation

**Fig. 1.**
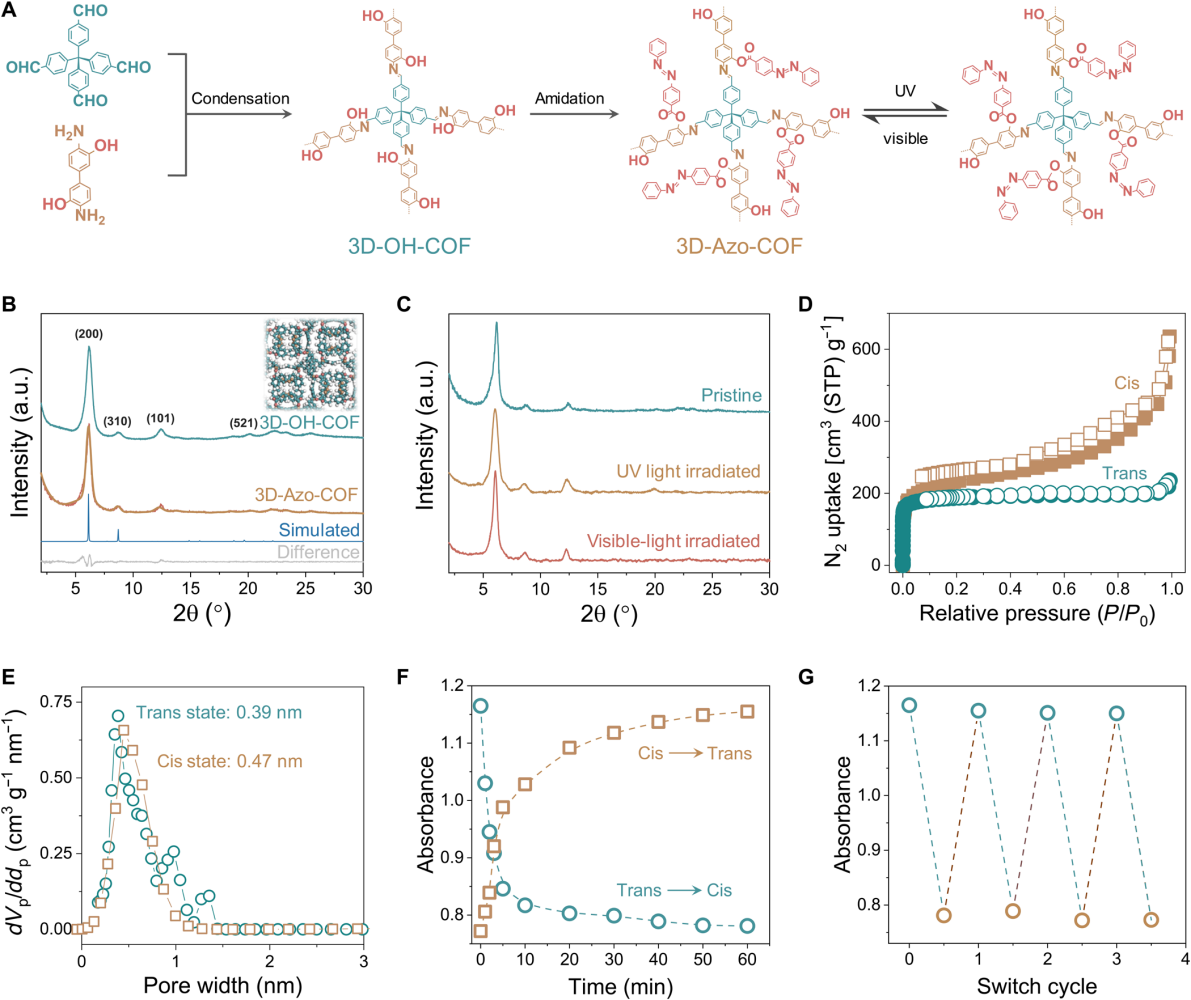
Synthesis and isomerization of 3D-Azo-COF. (**A**) Reaction sequence for post-synthetic modification of 3D-Azo-COF and its photoisomerization behavior. (**B**) Experimental PXRD patterns of 3D-OH-COF in cyan, 3D-Azo-COF in red, and simulated pattern of 3D-Azo-COF in blue, Pawley refinement in yellow, and difference between the experimental and simulated profiles in gray. Inset is the structural model of 3D-Azo-COF. (**C**) PXRD patterns of the 3D-Azo-COF upon the irradiation of UV light and visible light. (**D**) N_2_ sorption isotherms and (**E**) pore size distribution profiles of 3D-Azo-COF under trans and cis states. (**F**) Fitting curves of absorbance at 330 nm for 3D-Azo-COF. (**G**) Switching cycle of 3D-Azo-COF upon alternate irradiation at 365 and 450 nm. a.u., arbitrary unit.

To synthesize 3D COF suspended with azobenzene units, denoted as 3D-Azo-COF, we selected a diamine substituted with hydroxyl groups to give 3D-OH-COF, followed by the post-synthetic modification through the amidation reaction (Fig. 1A). The strategic incorporation of active center in the framework is vital for azobenzene grafting. Therefore, the chemical compositions and crystalline structures of 3D-OH-COF were firstly analyzed. A peak at 1619 cm^−1^ was detected from the Fourier transform infrared (FTIR) spectrum, attributing to the characteristic C═N stretching band (fig. S1). The stretching vibration peaks of C═O and N─H belonging to monomers were greatly attenuated after the condensation, which suggests that the monomers were consumed up. ^13^C nuclear magnetic resonance (NMR) spectrum exhibited a resonance signal at 131 parts per million (ppm), corresponding to the carbon atom of imine group, further confirming the formation of imine linkages (fig. S2). Powder x-ray diffraction (PXRD) revealed the formation of the desired crystalline framework (Fig 1B and fig. S3). The diffraction signals at 6.0°, 8.6°, 12.5°, and 20.5° are assigned to 200, 310, 101, and 521 reflections, which agree with those reported in literatures (*30*, *31*). Pawley refinement matches well with the observed profile, providing good agreement factors (*R*_p_ = 4.8% and *R*_wp_ = 6.5%) and indicating their negligible differences. The porosity of 3D-OH-COF was analyzed by N_2_ sorption test at 77 K. A rapid gas uptake at low relative pressures (*P*/*P*_0_ < 0.01) was observed, demonstrating its highly microporous property. Nonlocal density functional theory (NLDFT) fitting of the adsorption branch exhibits a narrow pore size distribution at 1.2 nm, in agreement with the proposed model (fig. S4).

The well-defined channel of 3D-OH-COF should allow the azobenzene groups inside accessible. We then performed the postsynthetic modification of the hydroxyl-tagged COF via amidation reaction, which is confirmed by the model reaction (fig. S5). The variation of the chemical composition was verified by FTIR, solid-state NMR, and x-ray photoelectron spectrometer (XPS) spectra. A new signal appears at 1650 cm^−1^, attributing to the N═N stretching mode. Solid-state NMR spectrum shows a characteristic peak at 138 ppm, which is ascribed to the aromatic carbons of the C─N═N─C unit. The O 1s spectrum of 3D-Azo-COF exhibits a strong peak at 351.6 eV, corresponding to the O in the carbonyl bond (fig. S6). These results confirm the graft of azobenzene groups into the 3D-OH-COF skeleton. The PXRD pattern of 3D-Azo-COF shows a diffraction pattern comparable to that of 3D-OH-COF with slightly broadened peak widths (Fig. 1B). This is attributed to the flexible azobenzene chains in the COF pores, which comprise its long-range order. Notably, Pawley refinement of the PXRD pattern reveals the probable structure in the eclipsed stacking mode, leading to good agreement factors (*R*_p_ = 2.95% and *R*_wp_ = 8.31%) and reasonable profile differences. Microporosity is retained after azobenzene grafting, with the Brunauer-Emmett-Teller (BET) surface area changed from 1262 to 735 m^2^ g^−1^, and its pore size varied to 0.39 nm (Fig. 1C). Adding long azobenzene chains is expected to occupy the pores of the 3D-OH-COF, as a result of increasing mass without a sufficient adsorption area. Besides, this also confirms the decoration of azobenzene groups and pore accessibility of 3D-Azo-COF after modification. High-resolution transmission electron microscopy (HRTEM) was used to image the crystal structure of 3D-Azo-COF (fig. S7). The lattice fringe with the interlayer distance of 0.38 nm corresponds to the (200) crystal plane, which matches well with the proposed structural model (fig. S8). Hexagonal arranged pores can be seen, giving direct evidence of these textured micropores in an order arrangement. Moreover, a fast Fourier transform reveals uniform distribution and symmetry of lattice fingers. Collectively, we can conclude that azobenzene groups are successfully incorporated into the 3D-OH-COF without disrupting its periodic structure, providing a great pore environment for subsequent photoisomerization.

As the azobenzene units adopt a nonlinear geometrical change during the photoisomerization, we first evaluated the effect of transform on the crystal structure of 3D-Azo-COF (Fig. 1C). Notably, the diffraction peaks are entirely retained after light exposure at 320 and 435 nm, as evident by their XRD patterns. This is attributed to the inherent pore interconnectivity of 3D COFs, guaranteeing the structural integrality during azobenzene isomerization (*32*, *33*). We further investigated the porous structures of 3D-Azo-COF after exposure to the UV light (Fig. 1D). The BET surface area increases to 822.6 m^2^ g^−1^, which is attributed to the collapsed structure of cis-state azobenzene, allowing more N_2_ molecules diffusing into the pores. Besides, change in pore size after UV light exposure was also noted, contributing to a diameter of 0.47 nm (Fig. 1E). The variation agrees with the result derived from the materials studio (fig. S9). With azobenzene groups in the trans-conformation, the pore size of the 3D-Azo-COF is calculated to be 0.37 nm, while it enhances to 0.48 nm after azobenzene groups converting to the cis-conformation. These results suggest that the pore channels of 3D-Azo-COF could be rational controlled upon external light, without comprising the structural regularity.

The photoisomerization process was further monitored by electronic absorption spectra (Fig. 1F). 3D-Azo-COF dispersed in tetrahydrofuran (THF) shows absorption bands at 330 and 450 nm, which are assigned to the π-π* and n-π* transitions, respectively (fig. S10). Upon UV light exposure, a synchronized increase in the π-π* transition and decrease in the n-π* transition was detected, suggesting the trans-to-cis isomerization of azobenzene units. Moreover, visible light triggers the reverse cis-to-trans isomerization within the same transition points (fig. S11). The variation of absorbance at 330 nm was further fitted using the first-order kinetic function, giving isomerization rate constants of 0.035 and 0.029 min^−1^ for trans-to-cis and cis-to-trans isomerization, respectively. This reveals the fast transformation and good reversibility of the conformation between trans- and cis-state azobenzene. The noticeable variation in the peak intensity after exposure for 2 min suggests a rapid response, enabling spatial and temporal controls of pores. Besides, negligible degradation in absorbance was observed even after four alternating light changes, indicating the robust photoisomerization for 3D-Azo-COF (Fig. 1G).

### Fabrication of 3D-Azo-COF membranes

**Fig. 2.**
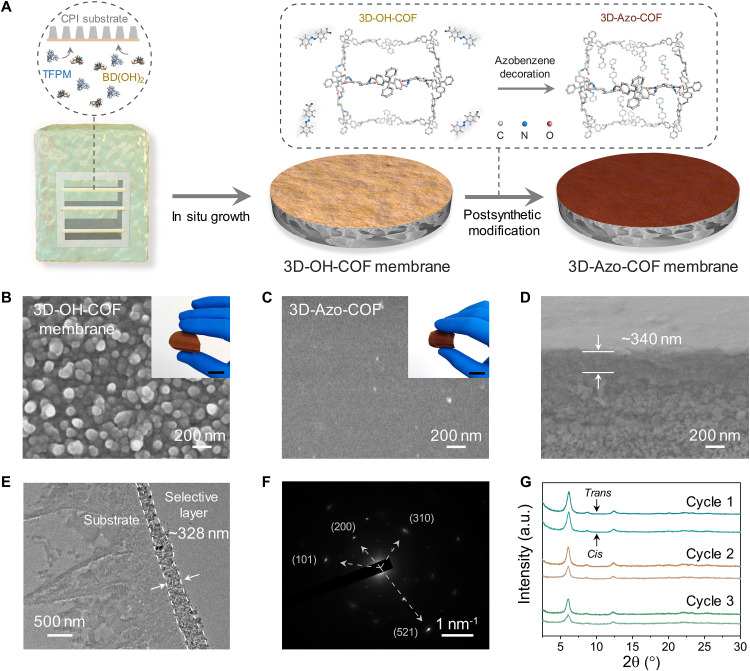
Fabrication and structural determination of 3D-Azo-COF membranes. (**A**) Schematic illustration of the fabrication of 3D-Azo-COF composite membranes. Surface SEM images of the (**B**) 3D-OH-COF and (**C**) 3D-Azo-COF membranes. Insets are the corresponding digital photos. (**D**) Cross-sectional SEM image of the 3D-Azo-COF membrane. (**E**) Cross-sectional TEM image and (**F**) the selected area electron diffraction of the 3D-Azo-COF membrane. (**G**) GIXRD patterns of the 3D-Azo-COF membrane subjected to cyclic photoisomerization with 365- and 450-nm lights for 1 hour. CPI, cross-linked polyimide; TFPM, tetra(4-formylphenyl)-methane; BD(OH)_2_, 3,3′-dihydroxybenzidine.

Having verified the photoactivity of 3D-Azo-COF, we then engineered 3D-OH-COF membranes on mesoporous cross-linked polyimide (CPI) supports via in situ growth, followed by the postsynthetic modification to produce 3D-Azo-COF membranes (Fig. 2A) (*31*). FTIR and XPS confirm the chemical conversion and presence of azobenzene groups (figs. S12 and S13). Top-view scanning electron microscopy (SEM) image demonstrates the continuous and cohesive surface morphology without defects for the 3D-OH-COF membrane (Fig. 2B). The crystallites intergrow and merge together in a packed manner, contributing to good mechanical strength. After amidation reaction, the surfaces of 3D-Azo-COF membranes become smooth compared with the pristine 3D-OH-COF membranes (Fig. 2C). The morphology evolution and the color change from yellow to brownness further suggest the graft of azobenzene groups. Control over membrane thickness was obtained by varying the concentrations of monomers, which can be finely tuned from 180 to 350 nm (Fig. 2D and fig. S14).

To detect the crystalline structure of the 3D-Azo-COF membrane, grazing incidence XRD (GIXRD) analyses were conducted using an incident angle of 0.5° to avoid the effect of supports (fig. S15). The 3D-OH-COF membrane exhibits distinct diffraction peaks consistent with the bulk powder. Note that the peak positions remain unchanged of the 3D-Azo-COF membrane, indicating the maintained crystalline skeleton during the post-synthetic modification process. HRTEM image exhibits that the crystalline 3D-Azo-COF adheres tightly to the amorphous CPI substrate, forming a seamless interface (Fig. 2E). This is ascribed to the great compatibility between the 3D-Azo-COF and the organic substrate. The diffraction dots showed by the 3D-Azo-COF layer correspond to the (200), (101), (310), and (521), respectively (Fig. 2F). These results match well with the structural model from the XRD pattern, suggesting the successful formation of the desired 3D-Azo-COF structure on the surface of the CPI substrate.

Light-activated structural stability of the membrane was further evaluated through systematic exposure to UV- and visible-light (Fig. 2G). The (200), (310), and (101) diffraction peaks are totally restored after trans-to-cis isomerization. This could be ascribed to the entire and strong covalent linkages of the 3D-Azo-COF membrane and the durability of decorated azobenzene units. Moreover, the structural isomerization could be stably conducted for three cycles without attenuation. Besides, peak force quantitative nanomechanical mapping (PFQNM) suggests that the 3D-Azo-COF membrane still has a decent mechanical strength after UV light exposure (fig. S16). This is attributed to the robust and permanent skeleton for maintaining the structural rigidity during the trans-to-cis isomerization. This favorable stability allows an effective and steady control of the membrane channel, which is of great importance for the long-term molecular separation.

### Gas transport properties

**Fig. 3.**
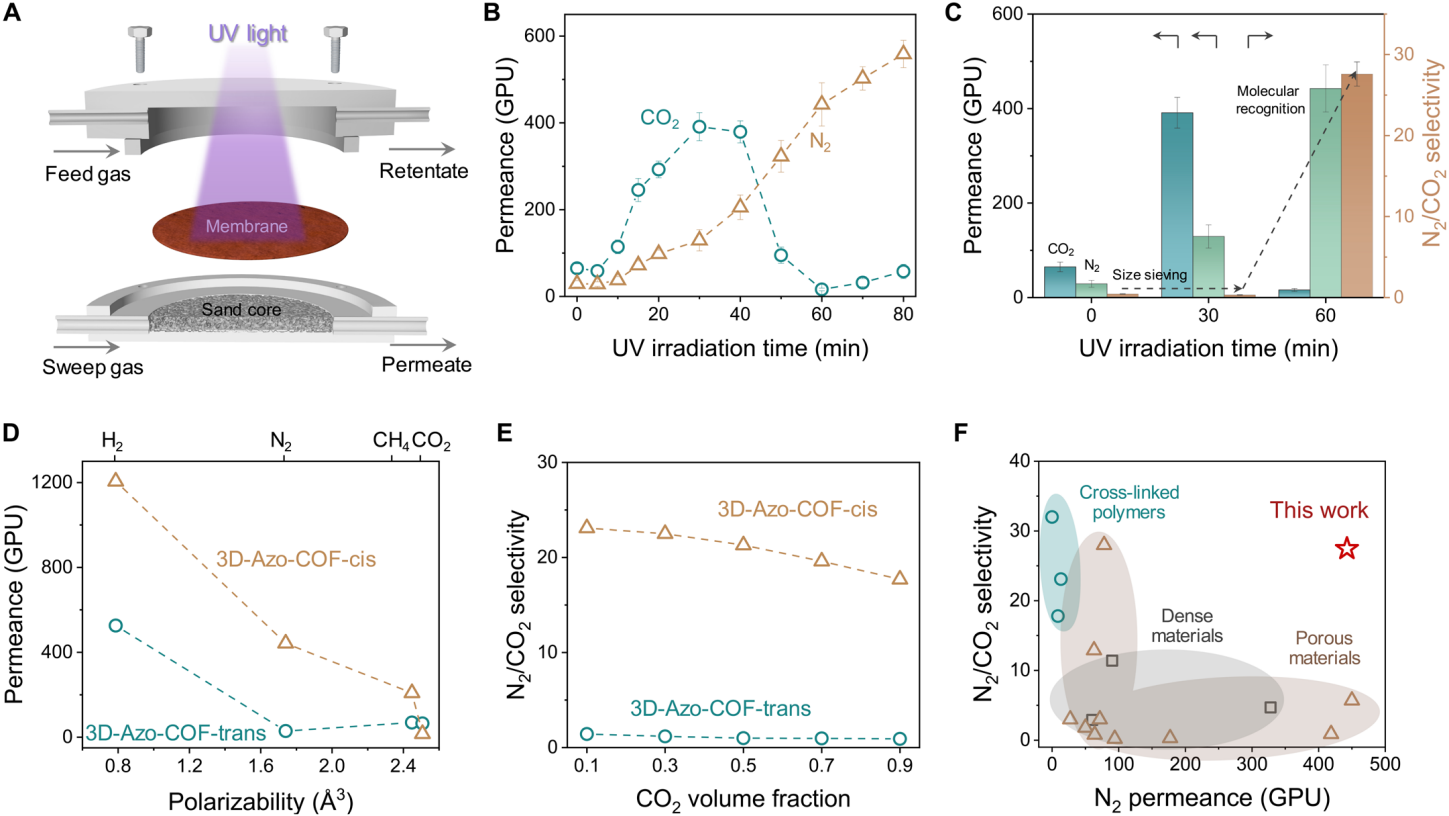
Light-activated N_2_/CO_2_ separation performances. (**A**) Sketch of the setup for the photoresponsive gas permeation tests. (**B**) UV-light controlled CO_2_ and N_2_ permeation as a function of irradiation time. (**C**) Comparison of N_2_/CO_2_ separation performances of the 3D-Azo-COF membrane under different irradiation time. (**D**) Gas transport behaviors of 3D-Azo-COF membranes exposure to UV light for 0 min (3D-Azo-COF-trans) and 60 min (3D-Azo-COF-cis). (**E**) Effect of the feed composition on the N_2_/CO_2_ separation performance of 3D-Azo-COF membranes. (**F**) Performance comparison of different kinds of membranes.

The permeation of gas molecules through the 3D-Azo-COF membrane was evaluated using a homemade apparatus (fig. S17). It can be seen from fig. S18 that CO_2_ and N_2_ permeances sharply decline with increased COF layer thickness. This is ascribed to the compact structure that endows enhanced gas transfer resistance. Specifically, the 3D-Azo-COF membrane with the thickness of 340 nm gives optimized N_2_ and CO_2_ permeances of 29.2 and 64.9 gas permeation unit (GPU), with the N_2_/CO_2_ selectivity of 0.45. To clarify the molecular affinity of the 3D-Azo-COF channels, we then investigated the gas transfer behaviors of this membrane subjected to UV light for varied time (Fig. 3A and fig. S19). When the irradiation time is prolonged to 30 min, the resulting CO_2_ permeance gradually increased to 391 GPU, whereas the N_2_ permeance simultaneously increased to 129 GPU (Fig. 3B). This is because of the enlarged pore size within the 3D-Azo-COF membrane after UV exposure, effectively promoting the molecular diffusion through the size-sieving effect. Notably, the rising rate of CO_2_ permeance is evidently higher than N_2_ permeance after UV exposure to 10 min, which may be ascribed to the favorable affinity between CO_2_ molecules and 3D-Azo-COF channels, promoting CO_2_ transport. Extending the irradiation time to 60 min leads to a sharp decline in the CO_2_ permeance. In contrast, the N_2_ permeance keeps rising to 443 GPU, presenting a monotonic increasing curve. These findings can be attributed to the sufficient trans-to-cis isomerization of the 3D-Azo-COF membrane, simultaneously enhancing the pore size and strengthening the interactivity between azobenzene groups and CO_2_ molecules. To further elucidate the variety of membrane transport properties with UV exposure time, we then compared the separation performance by analyzing the permeance-selectivity change tendency (Fig. 3C). Moderate UV exposure slightly open the pore channels, leading to fast CO_2_ transport based on the size-sieving effect. The molecular recognition effect starts to dominate after prolonging the UV exposure time, contributing to a high N_2_/CO_2_ ideal selectivity of 27.6. Moreover, the N_2_ recovery ratio is increased to 92.6% after exposure to UV light for 60 min. It indicates the high N_2_ enrichment efficiency of the 3D-Azo-COF membrane through the photoisomerization (fig. S20). These results demonstrates that rational control of the azobenzene isomerization is the prerequisite for realizing the molecular recognition effects, and both the size sieving and recognition effects are critical to the fabrication of CO_2_-selective channels.

With the trans-to-cis isomerization of azobenzene moieties, they can transform from the planar structure to the polar structure with a dipole moment of 3 D, leading to an obvious polarity enhancement (*34*). We then conducted permeation tests for the membrane under trans (3D-Azo-COF-trans) and cis (3D-Azo-COF-cis) states using single-component gases with different polarizabilities (Fig. 3D). The gases with low polarizability, such as H_2_ and N_2_, exhibit enhanced permeances for the 3D-Azo-COF-cis membrane, as the pore channels are opened upon UV exposure (table S1). In contrast, the declined CO_2_ permeance was detected despite having smaller molecular size, which is attributed to the high polarizability of CO_2_, contributing to strong binding force with the channels.

The CO_2_ flow rate plays a critical role on its separation performance as the surface interactions highly depend on the gas retention time within the membrane channels (*35*, *36*). To further elucidate the molecular recognition effect on CO_2_ transport behavior, we evaluated the gas separation performance using mixed N_2_/CO_2_ with different volume fractions (Fig. 3E). Keeping the feed pressure at 1 bar and the total flow rate at 100 ml min^−1^, the CO_2_ partial flow rate was varied from 10 to 90 ml min^−1^. With the increase of CO_2_ fraction, the N_2_/CO_2_ selectivity is basically unchanged for the 3D-Azo-COF-trans membrane, attributing to the size-sieving effect that works for the molecular separation. However, the effect of CO_2_ fraction on the selectivity becomes obvious for the 3D-Azo-COF-trans membrane. The elevated CO_2_ fraction leads to declined N_2_/CO_2_ selectivity from 23.1 to 17.7, which could be attributed to the less CO_2_-pore wall interaction. Besides, feed pressure is also a critical factor affecting the molecular transport behaviors (fig. S21). For the 3D-Azo-cis membrane, the feed flow rate was hold at 100 ml min^−1^ with equipotent gas fractions and varied transmembrane pressures. Increasing the pressure led to higher CO_2_ and N_2_ permeance, and meanwhile, the N_2_/CO_2_ selectivity gradually declined. It is notable that this effect shows negligible characteristics in the N_2_/CH_4_ separation system owing to their close polarizabilities (fig. S22). We further explored the impact of gas purity on the N_2_/CO_2_ selectivity of the 3D-Azo-COF-cis membrane (fig. S23). The selectivity slightly decreases with the introduction of 1% CO and NO_2_ that always involved in flue gas. This may be attributed to the competitive effect of CO_2_ and impurity gases, leading to a reduced CO_2_-pore wall interaction. These results suggest the importance of sufficient dipole-quadrupole interaction for recognizing CO_2_ molecules, which enables the efficient capture over N_2_.

Encouraged by the unique molecular transport behavior and decent structural stability, we sought to evaluate the durability of the 3D-Azo-COF membrane in the prolonged separation process. As illustrated in fig. S24, the gas permeance and the N_2_/CO_2_ selectivity both maintained almost unchanged during continuous operation for 72 hours. This demonstrates the great potential of our membrane in durable CO_2_ recognition and separation. We then studied the selectivity change after light activation (fig. S25). The N_2_/CO_2_ selectivity can be shifted between 21.1 and 1.3 upon UV and visible light exposure. Moreover, the separation can be reversibly and stably switched for three cycles, which is related to the switchable azobenzene polarity. This result further confirms the structural stability of the membrane, highlighting its potential for on-demand CO_2_ separation.

To elucidate the variety of gas transport properties with channel structure, the separation performance of a series of pure membranes is depicted on a permeance-selectivity trade-off (Fig. 3F and table S2). Although high selectivity is observed in polymer membranes, the randomly arranged chains lead to tedious transport pathway, compromising the gas transport permeance. Membranes prepared from some porous materials exhibit attractive N_2_ permeance and N_2_/CO_2_ selectivity but still suffer from considerable trade-off. In this work, we prove the feasibility of using UV light to precisely adjusting the pore size and polarity, which creates a favorable channel environment for discriminating CO_2_ and N_2_ molecules. As a result, our 3D-Azo-COF-cis membrane demonstrates a substantial advantage in both N_2_ permeance and selectivity, revealing its capacity for N_2_ enrichment.

### Molecular insights into transport mechanism

To gain molecular insights into the interactions between gas molecules and pore channels, DFT calculations were performed. UV light drives the azobenzene isomerization along the torsion path through the rotation of N═N─C angles (*37*). Consequently, we constructed three azobenzene molecules with different N═N─C angles of 120°, 180°, and 240° and termed as Azo-trans, Azo-half-cis, and Azo-cis, respectively (fig. S26). The computed interaction energy (∆*E*) for Azo-trans/CO_2_, Azo-half-cis/CO_2_, and Azo-cis/CO_2_ are −3.8, −13.1 and −19.2 kcal mol^−1^, respectively. It suggests that the azobenzene with cis structure strongly binds to CO_2_, while trans structure tends to allow the transport of CO_2_. Relatively, CO_2_ moderately binds to the azobenzene with half-isomerized structure, which may accelerate CO_2_ transport through reversible interaction, and corresponds to the UV light–induced permeation tests.

**Fig. 4.**
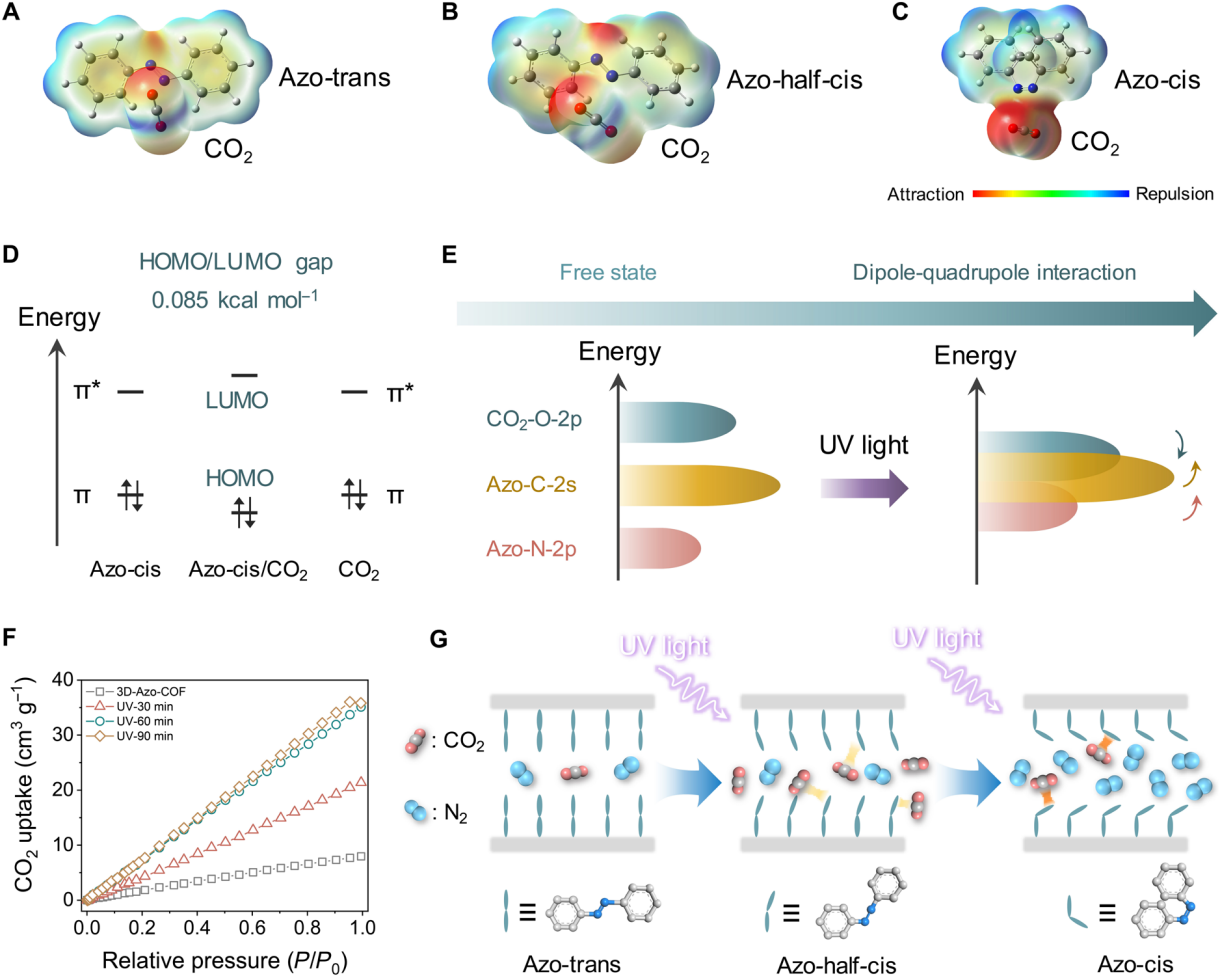
Molecular interactions between azobenzene and gases. The color-filled electrostatic complementary interactions between CO_2_ and azobenzene groups with (**A**) Azo-trans, (**B**) Azo-half-cis, and (**C**) Azo-cis states. The trend of red-blue represents the interactions quantified by an independent gradient model (−20 to 20 kcal mol^−1^). (**D**) Schematic atom interaction diagram of the Azo-cis/CO_2_ complex. (**E**) Orbital energy variation of atoms upon exposure to the UV light. (**F**) CO_2_ uptake isotherms at 298 K for 3D-Azo-COF powders upon different UV-light irradiation time. (**G**) Schematic showing the photo-regulated gas transport behaviors.

Electrostatic potential (ESP) maps were then performed to reveal the electron interaction in the complexes (Fig. 4, A to C). The negative ESP of azobenzene directly interacts with the C═O bond of CO_2_ having positive ESP. All three structures of azobenzene induce the electron hole of oxygen to be polarized. As a result, the mutual penetration extents of azobenzene with CO_2_ follow this order: Azo-trans < Azo-half-cis < Azo-cis, which is attributed to the strong electrostatic complementation within the Azo-cis/CO_2_ complex. This is coincident with the above binding strength between azobenzene and CO_2_, implying that the electrostatic interaction plays an important role in the combination of CO_2_.

Electronic structural nature of complexes was further studied using density of states (DOS) plots. From the partial DOS curves, the binding effect between azobenzene and CO_2_ is mainly contributed by C of azobenzene and O of CO_2_ (fig. S27). The frontier orbital gap, which is the gap between the lowest unoccupied molecular orbital (LUMO) and the highest occupied molecular orbital (HOMO), is determined to be 0.085 kcal mol^−1^ for the Azo-cis/CO_2_ complex (Fig. 4D). This HOMO-LUMO gap is smaller than Azo-trans/CO_2_ and Azo-half-cis/CO_2_ complexes, indicating its favorable electron transfer and stronger polarization with CO_2_. We then analyzed the fractional contributions of various atomic orbitals (fig. S28). Upon UV light exposure, the energies of C 2s and N 2p orbitals within azobenzene are increased, which lead to improved electron-donating ability (Fig. 4E). Besides, CO_2_ as an accepter obtains electrons from the O 2p orbital and shows declined energy. This result suggests that the 3D-Azo-COF channel has favorable electron affinity with CO_2_ and could strongly interact with CO_2_ under cis state.

Considering that the binding interaction is deeply related to the isomerization degree of azobenzene, we continued to expose 3D-Azo-COF powders to UV light for varied time. The sample was kept in the CO_2_ atmosphere, and in situ FTIR measurements were performed to dynamically monitor the structural evolution. UV light exposure led to the red shift of the N═N stretching band, indicating enhanced CO_2_-pore wall interaction (fig. S29) (*38*). Besides, the gas adsorption behaviors of these 3D-Azo-COF were further evaluated at 298 K (Fig. 4F). As expected, the CO_2_ uptake increases with the prolongation of UV light exposure time from 0 to 60 min. The extension of the irradiation time gives a slight increase in adsorption capacity, which may be attributed to complete isomerization of azobenzene side chains, leading to adsorption saturation. Notably, the uptake of CO_2_ is evidently higher than N_2_ for both samples after UV light exposure, owing to its higher polarizability (fig. S30).

According to the calculation and experimental results above, a schematic light-induced molecular transport behaviors is depicted in Fig. 4G. Both CO_2_ and N_2_ molecules freely pass through the 3D-Azo-COF channels because of their small kinetic diameters. Azobenzene undergoes trans-to-cis isomerization after UV light exposure, leading to slightly enlarged pore size and enhanced polarity of side chains. In this case, the membrane channels could reversibly bind with CO_2_ through the dipole-quadrupole interaction, which is attributes to the electron transfer between C═O of CO_2_ and C═N of azobenzene, greatly facilitating CO_2_ transport. Further intensifying the azobenzene isomerization contributes to enhanced electron attraction from azobenzene to CO_2_, which enables the pores with robust CO_2_ anchoring ability. Besides, the pore channel is fully enlarged and favors N_2_ molecules to access the membrane. Therefore, the 3D-Azo-COF-cis membrane has the strong binding sites that could easily recognize CO_2_, thereby favoring N_2_/CO_2_ separation.

## DISCUSSION

In summary, this work illustrates a 3D COF membrane featuring photosensitive azobenzene units onto the pore walls for discriminating CO_2_. Upon UV light activation, the 3D COF exhibits continuous conformational isomerization, with varied channel sizes and pore polarity. As a result, the 3D COF membrane receives different transport behaviors and mechanisms for different gas molecules. The increased intrapore dipole moment dominates the molecular transport and kinetics for CO_2_. In contrast, for the N_2_ molecule, the transport is controlled by the pore size, leading to a continuously enhanced permeance. DFT calculations suggest that the electron-donating ability of azobenzene is greatly enhanced after isomerizing to the cis-conformation, thus strengthening the CO_2_ trapping ability through the dipole-quadrupole interaction. Together with the size-sieving effect and a molecular recognition ability toward CO_2_, the 3D COF membrane contributes decent N_2_/CO_2_ selectivity with high N_2_ permeance. This work not only enables 3D COF membrane with adaptable pores but also paves the way for regulating gas transport kinetics, both of which will facilitate carbon capture and rational design of membranes for molecular separation.

## MATERIALS AND METHODS

### Materials

Tetra(4-formylphenyl)-methane (TFPM; 98%) and 3,3′-dihydroxybenzidine [BD(OH)_2_; 98%] were obtained from Jilin Chinese Academy of Sciences–Yanshen Technology Co. Ltd. 4-Phenylazobenzoyl chloride (95%) was purchased from Tokyo Chemical Industry Co. Ltd. Mesitylene (99%), 1,4-dioxane (99%), acetic acid (99%), THF (99%), methyl-2-pyrrolidinone (99%), ethyl acetate (99%), *n*-hexane (99%), phenol (99%), pyridine (99%), and poly(ethylene glycol) (weight-average molecular weight = 400 g mol^−1^; PEG400, 99%) were provided by Aladdin Co. Ltd. Isopropanol (99%) and hexanediamine (99%) were purchased from Macklin Co. Ltd. Polyimide powders were supplied by HP polymer Co. Ltd. H_2_, CO_2_, N_2_, CH_4_, and Ar with a minimum mole fraction of 99.999% for gas separation test were obtained from Nanjing Special Gases Company. Deionized water (DI water, conductivity: 2 to 8 μS cm^−1^) was used throughout this work. All materials were used without further purification.

### Preparation of 3D-Azo-COF membranes

The 3D-OH-COF membranes were prepared by in situ solvothermal growth on CPI substrates. In a typical process, TFPM (21.6 mg, 0.05 mmol) and BD(OH)_2_ (21.6 mg, 0.1 mmol) were weighed onto a 100-ml PTFE reactor, followed by addition of 18 ml of 1,4-dioxane and 2 ml of mesitylene. The mixture was sonicated for 20 min to obtain a homogeneous solution. CPI substrates pretreated with 1,4-dioxane and mesitylene (9:1, volume fraction) were placed into a home-made nylon holder and then immersed into the above solution. Subsequently, 500 μl of acetic acid was added into the mixture, and the reactor was sealed into a stainless-steel autoclave. The reaction mixture was heated at 85°C for 72 hours. After being cooled to room temperature, the obtained 3D-OH-COF membranes were washed with THF, 1,4-dioxane, and ethanol several times and stored in ethanol for further use.

The 3D-Azo-COF membranes were prepared using the amidation reaction. As-prepared 3D-OH-COF membranes were immersed in THF, and the exchange solvent was decanted and replenished three times. 4-Phenylazobenzoyl chloride (24 mg, 0.1 mmol) and pyridine (100 μl, 0.8 mmol) were dissolved in 20 ml of THF. Afterward, the home-made holder loaded with three pieces of 3D-OH-COF membranes was placed into the 100-ml PTFE reactor. The reactor was sealed and kept at 25°C for 24 hours. Last, the membranes were thoroughly washed with THF and ethanol to give 3D-Azo-COF membranes. The resultant membranes were kept in ethanol for further tests.

### DFT calculations

The structure of the azobenzene, CO_2_, and CO_2_-adsorbed azobenzene were all optimized using the density-fitting approximation-accelerated DFT of B3LYP functional, DFT-D3 dispersion correction method, and def2-SVP basis set. To obtain the electron energy with higher accuracy, single point calculations for these optimized structures with B3LYP functional and def2-TZVP basis set were performed. All these DFT calculations above were performed using ORCA 5.02 program. The binding energy between azobenzene and CO_2_ (∆*E*) was calculated from following equation

$$\text{∆}E=E_{\text{Azo}/{\text{CO}}_{2}}-E_{{\text{CO}}_{\text{2}}}-E_{\text{Azo}}$$

where *E*_Azo_, $E_{{\text{CO}}_{\text{2}}}$, and $E_{\text{Azo}/{\text{CO}}_{2}}$ represent the total energy for the azobenzene, CO_2_, and CO_2_ adsorbed azobenzene, respectively.

The charge density differences between azobenzene and CO_2_ were also calculated by simply subtracting the electron densities of azobenzene and CO_2_ from the electron density of complex structure using Multiwfn. The ESP and DOS were calculated using Multiwfn program and then rendered using GaussView program. The visualization of the frontier molecular orbitals and charge density difference were rendered using Visual Molecular Dynamic program.

## Supplementary Materials

### This PDF file includes:

Supplementary Text Figs. S1 to S30 Tables S1 and S2 References

## References

[1] C. G. Gruber, L. Frey, R. Guntermann, D. D. Medina, E. Cortés, Early stages of covalent organic framework formation imaged in operando. Nature 630, 872–877 (2024). 38839960 10.1038/s41586-024-07483-0 PMC11208157

[2] M. Feng, C. Xing, Y. Jin, X. Feng, Y. Zhang, B. Wang, Reticular chemistry for enhancing bioentity stability and functional performance. J. Am. Chem. Soc. 146, 32883–32905 (2024). 39561393 10.1021/jacs.4c09259

[3] J. Han, J. Feng, J. Kang, J.-M. Chen, X.-Y. Du, S.-Y. Ding, L. Liang, W. Wang, Fast growth of single-crystal covalent organic frameworks for laboratory x-ray diffraction. Science 383, 1014–1019 (2024). 38422145 10.1126/science.adk8680

[4] S. Li, S. Xu, E. Lin, T. Wang, H. Yang, J. Han, Y. Zhao, Q. Xue, P. Samorì, Z. Zhang, T. Zhang, Synthesis of single-crystalline sp^2^-carbon-linked covalent organic frameworks through imine-to-olefin transformation. Nat. Chem. 17, 226–232 (2025). 39762624 10.1038/s41557-024-01690-y

[5] W. Zhang, L. Chen, S. Dai, C. Zhao, C. Ma, L. Wei, M. Zhu, S. Y. Chong, H. Yang, L. Liu, Y. Bai, M. Yu, Y. Xu, X.-W. Zhu, Q. Zhu, S. An, R. S. Sprick, M. A. Little, X. Wu, S. Jiang, Y. Wu, Y.-B. Zhang, H. Tian, W.-H. Zhu, A. I. Cooper, Reconstructed covalent organic frameworks. Nature 604, 72–79 (2022). 35388196 10.1038/s41586-022-04443-4 PMC8986529

[6] X. Pang, B. Shi, Y. Liu, Y. Li, Y. Zhang, T. Wang, S. Xu, X. Wang, Z. Liu, N. Xing, X. Liang, Z. Zhu, C. Fan, Y. Liu, H. Wu, Z. Jiang, Phosphorylated covalent organic framework membranes toward ultrafast single lithium-ion transport. Adv. Mater. 36, e2413022 (2024). 39530691 10.1002/adma.202413022

[7] X. Tian, L. Cao, K. Zhang, R. Zhang, X. Li, C. Yin, S. Wang, Molecular weaving towards flexible covalent organic framework membranes for efficient gas separations. Angew. Chem. Int. Ed. 64, e202416864 (2025). 10.1002/anie.202416864 39377209

[8] A. Knebel, J. Caro, Metal-organic frameworks and covalent organic frameworks as disruptive membrane materials for energy-efficient gas separation. Nat. Nanotechnol. 17, 911–923 (2022). 35995854 10.1038/s41565-022-01168-3

[9] H. Yang, H. Zhang, C. Kang, C. Ji, D. Shi, D. Zhao, Solvent-responsive covalent organic framework membranes for precise and tunable molecular sieving. Sci. Adv. 10, eads0260 (2024). 39693424 10.1126/sciadv.ads0260 PMC11654673

[10] H. Fan, H. Wang, M. Peng, H. Meng, A. Mundstock, A. Knebel, J. Caro, Pore-in-pore engineering in a covalent organic framework membrane for gas separation. ACS Nano 17, 7584–7594 (2023). 37026681 10.1021/acsnano.2c12774 PMC10134499

[11] F. Yang, J. Guo, C. Han, J. Huang, Z. Zhou, S.-P. Sun, Y. Zhang, L. Shao, Turing covalent organic framework membranes via heterogeneous nucleation synthesis for organic solvent nanofiltration. Sci. Adv. 10, eadr9260 (2024). 39661688 10.1126/sciadv.adr9260 PMC11633759

[12] Y. Hu, B. Sengupta, H. Long, L. J. Wayment, R. Ciora, Y. Jin, J. Wu, Z. Lei, K. Friedman, H. Chen, M. Yu, W. Zhang, Molecular recognition with resolution below 0.2 angstroms through thermoregulatory oscillations in covalent organic frameworks. Science 384, 1441–1447 (2024). 38935724 10.1126/science.adj8791

[13] Y. Zhao, X. Tao, J. Lin, S. Lin, Azobenzene functionalized organic covalent frameworks: Controlled morphologies and photo-regulated adsorption. Adv. Funct. Mater. 33, 2302225 (2023).

[14] F. Auras, L. Ascherl, V. Bon, S. M. Vornholt, S. Krause, M. Döblinger, D. Bessinger, S. Reuter, K. W. Chapman, S. Kaskel, R. H. Friend, T. Bein, Dynamic two-dimensional covalent organic frameworks. Nat. Chem. 16, 1373–1380 (2024). 38702406 10.1038/s41557-024-01527-8

[15] C. Yin, L. Liu, Z. Zhang, Y. Du, Y. Wang, Photo-induced geometry and polarity gradients in covalent organic frameworks enabling fast and durable molecular separations. Small 20, 2309329 (2024). 10.1002/smll.202309329 38221705

[16] Y. Li, B. Xue, J. Yang, J. Jiang, J. Liu, Y. Zhou, J. Zhang, M. Wu, Y. Yuan, Z. Zhu, Z. J. Wang, Y. Chen, Y. Harabuchi, T. Nakajima, W. Wang, S. Maeda, J. P. Gong, Y. Cao, Azobenzene as a photoswitchable mechanophore. Nat. Chem. 16, 446–455 (2023). 38052946 10.1038/s41557-023-01389-6

[17] F. A. Jerca, V. V. Jerca, R. Hoogenboom, Advances and opportunities in the exciting world of azobenzenes. Nat. Rev. Chem. 6, 51–69 (2022). 37117615 10.1038/s41570-021-00334-w

[18] J. Liu, S. Wang, T. Huang, P. Manchanda, E. Abou-Hamad, S. P. Nunes, Smart covalent organic networks (CONs) with “on-off-on” light-switchable pores for molecular separation. Sci. Adv. 6, eabb3188 (2020). 32875111 10.1126/sciadv.abb3188 PMC7438094

[19] C. Yin, Z. Zhang, Z. Si, X. Shi, Y. Wang, Smart covalent organic frameworks with intrapore azobenzene groups for light-gated ion transport. Chem. Mater. 34, 9212–9220 (2022).

[20] G. Das, T. Prakasam, M. A. Addicoat, S. K. Sharma, F. Ravaux, R. Mathew, M. Baias, R. Jagannathan, M. A. Olson, A. Trabolsi, Azobenzene-equipped covalent organic framework: Light-operated reservoir. J. Am. Chem. Soc. 141, 19078–19087 (2019). 31656067 10.1021/jacs.9b09643

[21] G. Wang, Y. Feng, X. Ye, Z. Li, S. Tao, D. Jiang, Light-gating crystalline porous covalent organic frameworks. J. Am. Chem. Soc. 146, 10953–10962 (2024). 38565222 10.1021/jacs.4c02164

[22] D. Urban, N. Marcucci, C. H. Wölfle, J. Torgersen, D. R. Hjelme, E. Descrovi, Polarization-driven reversible actuation in a photo-responsive polymer composite. Nat. Commun. 14, 6843 (2023). 37891157 10.1038/s41467-023-42590-y PMC10611746

[23] Q. Huang, Z. Zhan, R. Sun, J. Mu, B. Tan, C. Wu, Light triggered pore size tuning in photoswitching covalent triazine frameworks for low energy CO_2_ capture. Angew. Chem. Int. Ed. 62, e202305500 (2023). 10.1002/anie.202305500 37162131

[24] Y. Feng, G. Wang, R. Liu, X. Ye, S. Tao, M. A. Addicoat, Z. Li, Q. Jiang, D. Jiang, Photoresponsive covalent organic frameworks: Visible-light controlled conversion of porous structures and its impacts. Angew. Chem. Int. Ed. 63, e202400009 (2024). 10.1002/anie.202400009 38415815

[25] Y. Yin, Y. Zhang, X. Zhou, B. Gui, W. Wang, W. Jiang, Y.-B. Zhang, J. Sun, C. Wang, Ultrahigh–surface area covalent organic frameworks for methane adsorption. Science 386, 693–696 (2024). 39509500 10.1126/science.adr0936

[26] F. Jin, E. Lin, T. Wang, S. Geng, L. Hao, Q. Zhu, Z. Wang, Y. Chen, P. Cheng, Z. Zhang, Rationally fabricating three-dimensional covalent organic frameworks for propyne/propylene separation. J. Am. Chem. Soc. 144, 23081–23088 (2022). 36484259 10.1021/jacs.2c10548

[27] F. Chen, H. Zheng, Y. Yusran, H. Li, S. Qiu, Q. Fang, Exploring high-connectivity three-dimensional covalent organic frameworks: Topologies, structures, and emerging applications. Chem. Soc. Rev. 54, 484–514 (2025). 39585733 10.1039/d4cs00703d

[28] Y. Xie, W. Wang, Z. Zhang, J. Li, B. Gui, J. Sun, D. Yuan, C. Wang, Fine-tuning the pore environment of ultramicroporous three-dimensional covalent organic frameworks for efficient one-step ethylene purification. Nat. Commun. 15, 3008 (2024). 38589420 10.1038/s41467-024-47377-3 PMC11001888

[29] X. Guan, F. Chen, S. Qiu, Q. Fang, Three-dimensional covalent organic frameworks: From synthesis to applications. Angew. Chem. Int. Ed. 62, e202213203 (2022). 10.1002/anie.202213203 36253336

[30] Q. Lu, Y. Ma, H. Li, X. Guan, Y. Yusran, M. Xue, Q. Fang, Y. Yan, S. Qiu, V. Valtchev, Postsynthetic functionalization of three-dimensional covalent organic frameworks for selective extraction of lanthanide ions. Angew. Chem. Int. Ed. 57, 6042–6048 (2018). 10.1002/anie.201712246 29457858

[31] X. Shi, Z. Zhang, S. Fang, J. Wang, Y. Zhang, Y. Wang, Flexible and robust three-dimensional covalent organic framework membranes for precise separations under extreme conditions. Nano Lett. 21, 8355–8362 (2021). 34596413 10.1021/acs.nanolett.1c02919

[32] S. E. Neumann, J. Kwon, C. Gropp, L. Ma, R. Giovine, T. Ma, N. Hanikel, K. Wang, T. Chen, S. Jagani, R. O. Ritchie, T. Xu, O. M. Yaghi, The propensity for covalent organic frameworks to template polymer entanglement. Science 383, 1337–1343 (2024). 38513024 10.1126/science.adf2573

[33] R. M. Zhu, Y. Liu, W. K. Han, J. D. Feng, J. Zhang, H. Pang, J. Zhang, Z. G. Gu, Three-dimensional covalent organic frameworks based on linear and trigonal linkers for high-performance H_2_O_2_ photosynthesis. Angew. Chem. Int. Ed. 64, e202412890 (2024). 10.1002/anie.202412890 39148428

[34] J. Gemen, J. R. Church, T.-P. Ruoko, N. Durandin, M. J. Białek, M. Weißenfels, M. Feller, M. Kazes, M. Odaybat, V. A. Borin, R. Kalepu, Y. Diskin-Posner, D. Oron, M. J. Fuchter, A. Priimagi, I. Schapiro, R. Klajn, Disequilibrating azobenzenes by visible-light sensitization under confinement. Science 381, 1357–1363 (2023). 37733864 10.1126/science.adh9059

[35] L. Cheng, Y. Guo, Q. Liu, G. Liu, R. Li, X. Chen, H. Zeng, G. Liu, W. Jin, Metal confined in 2D membranes for molecular recognition and sieving towards ethylene/ethane separation. Adv. Mater. 34, 2206349 (2022). 10.1002/adma.202206349 36039875

[36] R. Yang, Y. Wang, J.-W. Cao, Z.-M. Ye, T. Pham, K. A. Forrest, R. Krishna, H. Chen, L. Li, B.-K. Ling, T. Zhang, T. Gao, X. Jiang, X.-O. Xu, Q.-H. Ye, K.-J. Chen, Hydrogen bond unlocking-driven pore structure control for shifting multi-component gas separation function. Nat. Commun. 15, 804 (2024). 38280865 10.1038/s41467-024-45081-w PMC10821866

[37] J. K. Yu, C. Bannwarth, R. Liang, E. G. Hohenstein, T. J. Martinez, Nonadiabatic dynamics simulation of the wavelength-dependent photochemistry of azobenzene excited to the nπ* and ππ* excited states. J. Am. Chem. Soc. 142, 20680–20690 (2020). 33228358 10.1021/jacs.0c09056

[38] Q. Xu, J. Han, F. Tian, X. Zhao, J. Rong, J. Zhang, P. She, J.-S. Qin, H. Rao, Synergistic bifunctional covalent organic framework for efficient photocatalytic CO_2_ reduction and water oxidation. J. Am. Chem. Soc. 147, 10587–10597 (2025). 40071963 10.1021/jacs.5c00432

[39] I. C. D. Merritt, D. Jacquemin, M. Vacher, *cis* → *trans* photoisomerisation of azobenzene: A fresh theoretical look. Phys. Chem. Chem. Phys. 23, 19155–19165 (2021). 34195720 10.1039/d1cp01873f

[40] T. Ashirov, J. S. Siena, M. Zhang, A. Ozgur Yazaydin, M. Antonietti, A. Coskun, Fast light-switchable polymeric carbon nitride membranes for tunable gas separation. Nat. Commun. 13, 7299 (2022). 36435832 10.1038/s41467-022-35013-x PMC9701225

[41] H. Li, L. Han, J. Hou, J. Liu, Y. Zhang, Oriented zeolitic imidazolate framework membranes within polymeric matrices for effective N_2_/CO_2_ separation. J. Membr. Sci. 572, 82–91 (2019).

[42] Y. Zhou, Y. Wu, H. Wu, J. Xue, L. Ding, R. Wang, H. Wang, Fast hydrogen purification through graphitic carbon nitride nanosheet membranes. Nat. Commun. 13, 5852 (2022). 36195763 10.1038/s41467-022-33654-6 PMC9532387

[43] Y. Ying, S. B. Peh, H. Yang, Z. Yang, D. Zhao, Ultrathin covalent organic framework membranes via a multi-interfacial engineering strategy for gas separation. Adv. Mater. 34, e2104946 (2022). 34535914 10.1002/adma.202104946

[44] H. Guo, G. Zhu, I. J. Hewitt, S. Qiu, “Twin Copper Source” growth of metal−organic framework membrane: Cu_3_(BTC)_2_ with high permeability and selectivity for recycling H_2_. J. Am. Chem. Soc. 131, 1646–1647 (2009). 19159223 10.1021/ja8074874

[45] H. Fan, A. Mundstock, A. Feldhoff, A. Knebel, J. Gu, H. Meng, J. Caro, Covalent organic framework-covalent organic framework bilayer membranes for highly selective gas separation. J. Am. Chem. Soc. 140, 10094–10098 (2018). 30021065 10.1021/jacs.8b05136

[46] G. Xu, J. Yao, K. Wang, L. He, P. A. Webley, C.-s. Chen, H. Wang, Preparation of ZIF-8 membranes supported on ceramic hollow fibers from a concentrated synthesis gel. J. Membr. Sci. 385-386, 187–193 (2011).

[47] Z. Zhong, J. Yao, R. Chen, Z. Low, M. He, J. Z. Liu, H. Wang, Oriented two-dimensional zeolitic imidazolate framework-L membranes and their gas permeation properties. J. Mater. Chem. A 3, 15715–15722 (2015).

[48] K. Kgaphola, I. Sigalas, M. O. Daramola, Synthesis and characterization of nanocomposite SAPO-34/ceramic membrane for post-combustion CO_2_ capture. Asia Pac. J. Chem. Eng. 12, 894–904 (2017).

[49] W. Li, G. Zhang, C. Zhang, Q. Meng, Z. Fan, C. Gao, Synthesis of trinity metal–organic framework membranes for CO_2_ capture. Chem. Commun. 50, 3214–3216 (2014). 10.1039/c3cc49815h 24522328

[50] P. Kumar, S. Kim, J. Ida, V. V. Guliants, Polyethyleneimine-modified MCM-48 membranes: Effect of water vapor and feed concentration on N_2_/CO_2_ selectivity. Ind. Eng. Chem. Res. 47, 201–208 (2008).

[51] K. Weh, M. Noack, K. Hoffmann, K. P. Schröder, J. Caro, Change of gas permeation by photoinduced switching of zeolite-azobenzene membranes of type MFI and FAU. Micropor. Mesopor. Mater. 54, 15–26 (2002).

[52] T. G. Kwon, J. Lee, O. H. Jo, B. G. Kang, S. W. Kang, Charged surface of polyacrylonitrile colloid and its application to N_2_/CO_2_ separation. Macromol. Chem. Phys. 225, 2300323 (2023).

[53] J. Lee, H. Sohn, S. W. Kang, Surface of CuO nanoparticles modified by p-benzoquinone for N_2_-selective membrane. Membranes 12, 1229 (2022). 36557136 10.3390/membranes12121229 PMC9787012

[54] S. Yu, B. Qin, F. Yang, M. Xie, L. Xue, Z. Zhao, K. Wang, Unlocking the limits of diffusion and adsorption of metal-crosslinked reduced graphene oxide membranes for gas separation. Appl. Surf. Sci. 586, 152868 (2022).

[55] J. Shen, G. Liu, Y. Ji, Q. Liu, L. Cheng, K. Guan, M. Zhang, G. Liu, J. Xiong, J. Yang, W. Jin, 2D mxene nanofilms with tunable gas transport channels. Adv. Funct. Mater. 28, 1801511 (2018).

[56] F. A. Nezhad, N. Han, Z. Shen, Y. Jin, Y. Wang, N. Yang, S. Liu, Experimental and theoretical exploration of gas permeation mechanism through 2D graphene (not graphene oxides) membranes. J. Membr. Sci. 601, 117883 (2020).

