# Supplementary material for: Light-activated 3D covalent organic framework membranes with adaptive pores for CO2 recognition and separation
Source: Sci Adv. 2025 Aug 6;11(32):eadw8452. doi: 10.1126/sciadv.adw8452 (PMC12327475; doi:10.1126/sciadv.adw8452)
Supplement: Supplementary file 1 — Supplementary Text Figs. S1 to S30 Tables S1 and S2 References [file sciadv.adw8452_sm.pdf]

Science Advances
AAAS

Supplementary Materials for

Light-activated 3D covalent organic framework membranes with adaptive pores for CO_2 recognition and separation

Congcong Yin *et al.*

Corresponding author: Yong Wang, yongwang@seu.edu.cn

Sci. Adv. **11**, eadw8452 (2025)
DOI: 10.1126/sciadv.adw8452

This PDF file includes:

Supplementary Text
Figs. S1 to S30
Tables S1 and S2
References

Synthesis of 3D-OH-COF powders

3D-OH-COF was synthesized using the previously reported procedure. A Pyrex tube was charged with TFPM (21.6 mg, 0.05 mmol), $BD(OH)_2$ (21.6 mg, 0.1 mmol), 1,4-dioxane (0.9 mL), mesitylene (0.1 mL) and 3 M aqueous acetic acid (0.1 mL). The mixture was sonicated for 10 min, and the tube was then flash-frozen at 77.3 K and degassed by three freeze-pump-thaw cycles. The tube was sealed and heated at 120°C without disturbance for 72 h. After being cooled to room temperature, the precipitates were isolated by filtration. To activate the 3D-OH-COF, solvent exchange was performed with THF, in which the powder was immersed in THF and the solvent was removed and replenished three times. The product was then dried under vacuum at 60°C overnight to yield a yellow solid (35 mg, yield 81%).

Synthesis of 3D-Azo-COF powders

In the suspension of 3D-OH-COF (30 mg) in THF (5 mL), pyridine (100 μL, 0.8 mmol), 4-phenylazobenzoyl chloride (24 mg, 0.1 mmol) were added. The mixture was placed at 25°C under the N_2 atmosphere for 24 h with vigorous stirring. The crude solids were acquired by filtration and washed with THF, and further exhaustively washed by Soxhlet extractions with THF for 24 h. The resultant brown powders were dried at 80°C under vacuum overnight.

Synthesis of model compound

Phenol (14 μL, 0.15 mmol) and pyridine (100 μL, 0.8 mmol) serving as the acid scavenger were charged into a pre-dried flask containing 5 mL of THF. Subsequently, 4-phenylazobenzoyl chloride (24 mg, 0.1 mmol) dissolved in 5 mL of THF was dropwise added into the mixture under vigorous stirring. Then the reaction mixture was stirred for 6 h at 25°C. The product was purified on a silica gel column and eluted with ethyl acetate/n-hexane (1:4) to give the product (26 mg, yield 68%).

Preparation of cross-linked polyimide (CPI) substates

Non-solvent induced phase separation (NIPS) was used for preparing PI substrates. To obtain the casting solution with a concentration of 20 wt%, PI powders were dissolved into a mixture containing 93.75 wt% NMP and 6.25% PEG400, followed by mechanical stirring at 60ºC for ample time. The polymer dope was left in vacuum overnight to remove excessive air bubbles. Subsequently, the obtained solution was casted onto a nonwoven support using a casting knife with a gate height of 200 μm, which was immediately immersed into the coagulation bath of DI water for solvent-nonsolvent exchange. The PI substrates were thoroughly washed with DI water. After that, the resultant PI substrates were rinsed with isopropanol at least for three times and kept in isopropanol for further use.

To obtain the CPI substrates, the prepared PI substrates were soaked into the isopropanol solution containing 5 wt% hexanediamine for 24 h at room

temperature. Whereafter, the CPI substrates were rinsed with isopropanol to thoroughly remove the residual hexanediamine and stored in isopropanol.

Characterizations

Fourier transform infrared spectroscopy (FTIR) spectra were recorded on neat samples in the range of 3500–1200 cm^{-1} on a Nicolet 8700 spectrometer (Thermo Fisher Scientific). Powder samples were prepared by potassium bromide tablets, and the membrane samples were tested using the attenuated total reflectance (ATR) mode. In situ FT-IR measurements were conducted using the Bruker INVENIO R FT-IR spectrometer equipped with an in situ diffuse reflectance cell (Harrick). The ^{1}H spectrum of the model compound was recorded on a JNM-ECZ400S NMR spectrometer. Solid-state ^{13}C nuclear magnetic resonance (NMR) spectra were performed on an Agilent 600 DD2 spectrometer at a resonance frequency of 150.72 MHz under magic-angle spinning condition. X-ray photoelectron spectroscopy (XPS) data were measured in membrane form using a Thermo Scientific K-Alpha instrument with a monochromatized Al Kα line source. X-ray diffraction (XRD) patterns were collected on a Rigaku SmartLab diffractometer with Cu Kα radiation (λ = 0.15418 nm) at 40 kV and 30 mA. Data were recorded in the 2θ range of 0-30° with the scanning rate of 0.01° s^{-1}. The surface areas of 3D COFs were measured by nitrogen adsorption at 77.3 K using a BELSORP MAX adsorption analyser. For the low-pressure gas adsorption measurements of CO_2 and N_2,

the tests were performed at 298.15 K and the corresponding saturated vapor pressure was set as 100 kPa. Before the above adsorption-desorption analyses, the samples were sealed into glass tubes in the dark environment, and degassed offline at 120ºC for at least 12 h under a dynamic vacuum (10^{-5} bar). The specific surface areas were assessed using the Brunauer-Emmett-Teller (BET) model. Pore size distributions of 3D COFs were evaluated from fitting the nonlocal density functional theory (NLDFT) (cylindrical pore model) to the adsorption data. For the low-pressure gas adsorption measurements of CO_2, the tests were performed at 298.15 K and the corresponding saturated vapor pressure was set as 100 kPa. Thermogravimetric analyses were performed on an TGA2 (Mettler-Toledo) by heating samples at 10°C min^{-1} under a nitrogen atmosphere to 900ºC. High-resolution transmission electron microscopy (HRTEM) images of 3D COF powders were acquired using JEOL JEM-F200 under the operation voltage of 200 kV. The TEM samples were prepared by dropping the 3D COF dispersion onto a copper grid with carbon supporting film and dried under infrared ovens lamp. Field emission scanning electron microscopy (SEM, Hitachi S-4800, Japan) was used to observe the membrane surface morphologies and thickness at an accelerating voltage of 3 kV and a current of 10 μA. Before tests, the samples were sputter-coated with a thin layer of gold to enhance their conductivity. The Young's moduli were evaluated using the peak force quantitative nanomechanical mapping (PFQNM) mode at 1 kHz (Bruker Dimension ICON). Irradiation tests were conducted with a xenon lamp

CELHXF300-T3 (China Education Au-Light Ltd.). Irradiations with the wavelength of 365 and 450 nm were filtered using the optical filter. The light source was placed at 5 cm from the sample to keep the constant power density.

Gas permeation experiments

Based on the Wicke-Kallenbach method, single gas and mixed gas permeation tests were performed to evaluate the gas separation performance of 3D-Azo-COF membranes using a constant pressure technique. The membranes were sealed in a home-made module, and the effective permeation area was about 0.5 cm^2. The operating temperature was kept at 25°C to avoid thermal-induced isomerization. The membranes were exposure to 365 and 450 nm lights for varied durations. Prior to the permeation experiments, the entire system was evacuated for 30 min in order to remove absorbed atmospheric gases, and kept in an oven with a dark environment and constant temperature of 25°C(*39*). For the single gas permeation test, the flow rate of the feed gases was set as 30 mL min^{-1}. When the system reached a steady state, the permeance experiment of each gas was performed at least for three times. The gas permeance was calculated using the following equation:

$$P = \frac{1}{\Delta p\ A} \frac{273.15}{273.15 + T} (\frac{d_V}{d_t})$$

where *P* is the gas permeance (GPU, 1 GPU= 10^{-6} cm^3 (STP) cm^{-2} s^{-1} $cmHg^{-1}$), Δp is the transmembrane pressure (cmHg), *A* is the effective permeation area (cm^2), *T* is the operation temperature (°C), and d_V/d_t is the volumetric

displacement rate in the bubble flow meter.

The ideal selectivity of gas i to gas j, $\alpha_{i/j}$, was determined by the ratio of the permeance of the individual gases which can be presented as follows:

$$\alpha_{i/j} = \frac{P_i}{P_j}$$

where P_i and P_j denote the permeance of gases i and j, respectively.

A binary gas mixture of CO_2/N_2 (1:1; 1:3; 1:5; 1:7; 1:9, volume fraction) was used for mixed gas permeation tests. The mixed gas permeation experiments were carried out using the modified single-gas permeation apparatus integrated with gas chromatography (Panna A91Plus). Ar with the flow rate of 50 mL min^{-1} was chosen as the sweep gas. The separation factor for N_2/CO_2 can be calculated using the following equation:

$$\alpha_{N_2/CO_2} = \frac{y_{N_2}/y_{CO_2}}{x_{N_2}/x_{CO_2}}$$

where x and y are the molar fractions of the N_2 or CO_2 in the feed and permeate side, respectively.

The recovery ratio (R_i) of component i was calculated using the following equation:

$$R_i = \frac{y_i F_P}{x_i F_f} \times 100\%$$

where F_p and F_f represent the flow rate of permeate and feed, respectively.

The mixed gas permeance of component i was determined by the following equation:

$$P_i = \frac{1}{\Delta p\ A} \frac{273.15}{273.15 + T} \left(\frac{d_V}{d_t}\right) y_i$$

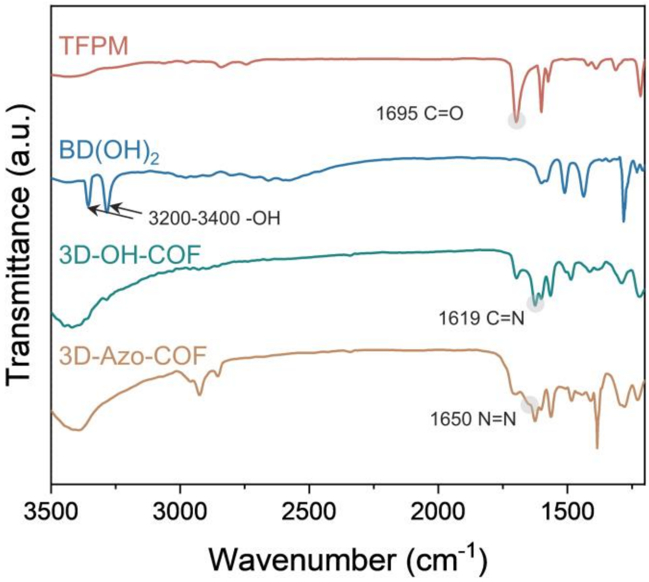

Fig. S1. FTIR spectra of TFPM and $BD(OH)_2$ monomers, 3D-OH-COF and 3D-Azo-COF powders.

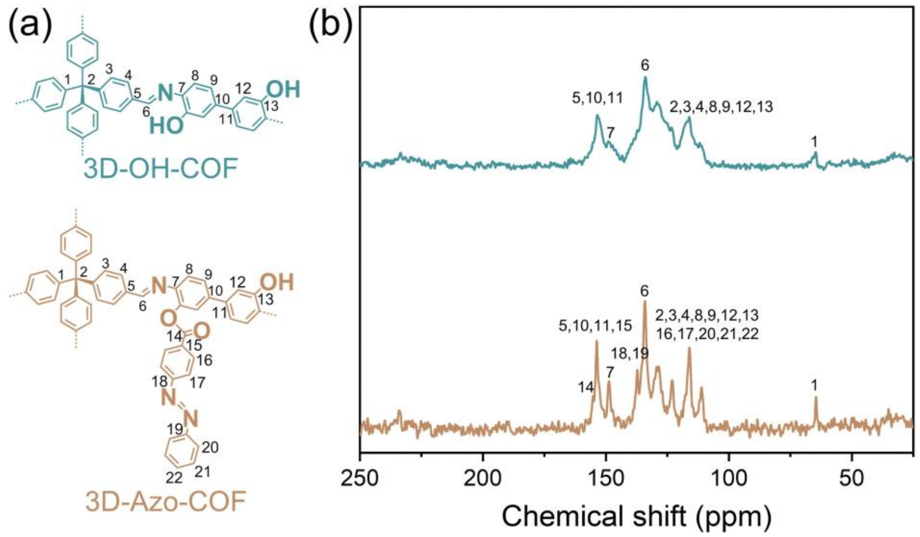

Fig. S2. (a) Chemical structures and (b) solid-state ^{13}C NMR spectra of 3D-OH-COF and 3D-Azo-COF.

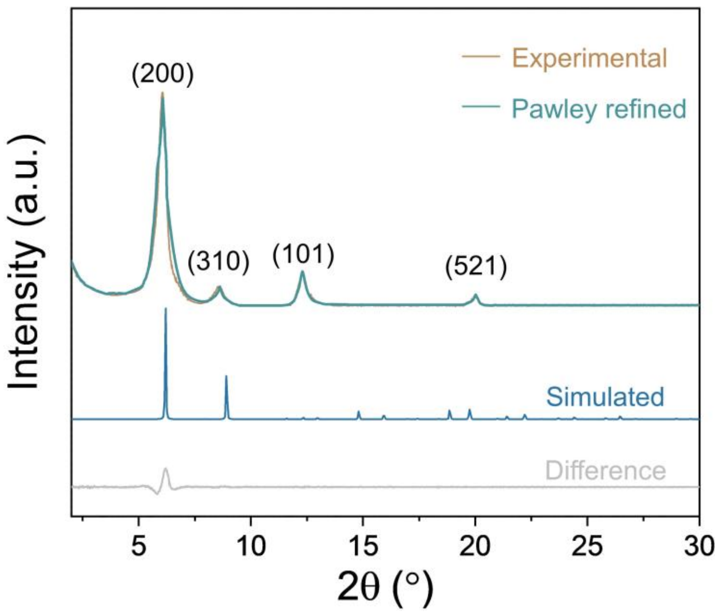

Fig. S3. Experimental PXRD patterns of 3D-OH-COF in yellow, and simulated pattern in blue, Pawley refinement in cyan, difference between the experimental and simulated profiles in gray.

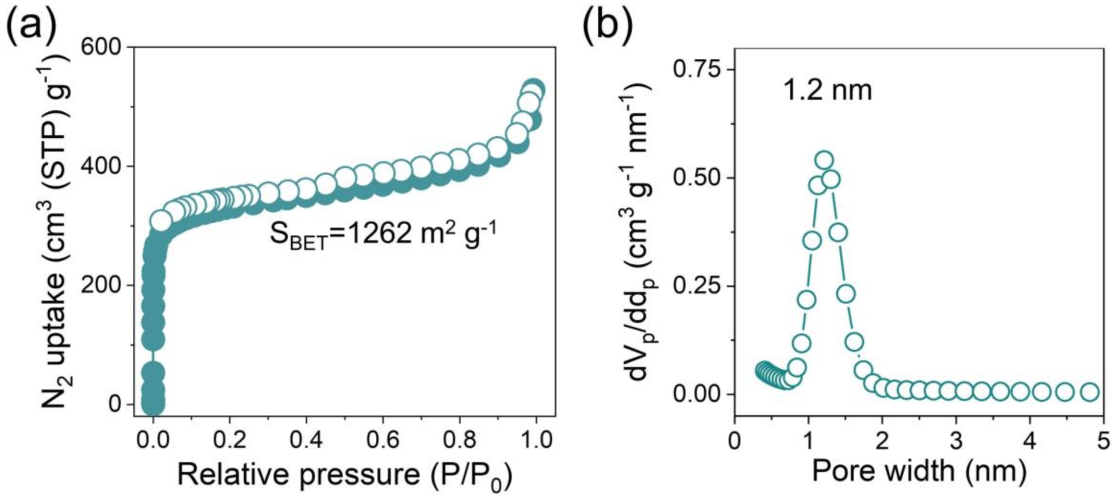

Fig. S4. (a) N_2 sorption isotherm (b) and the corresponding pore size distribution profile of 3D-OH-COF.

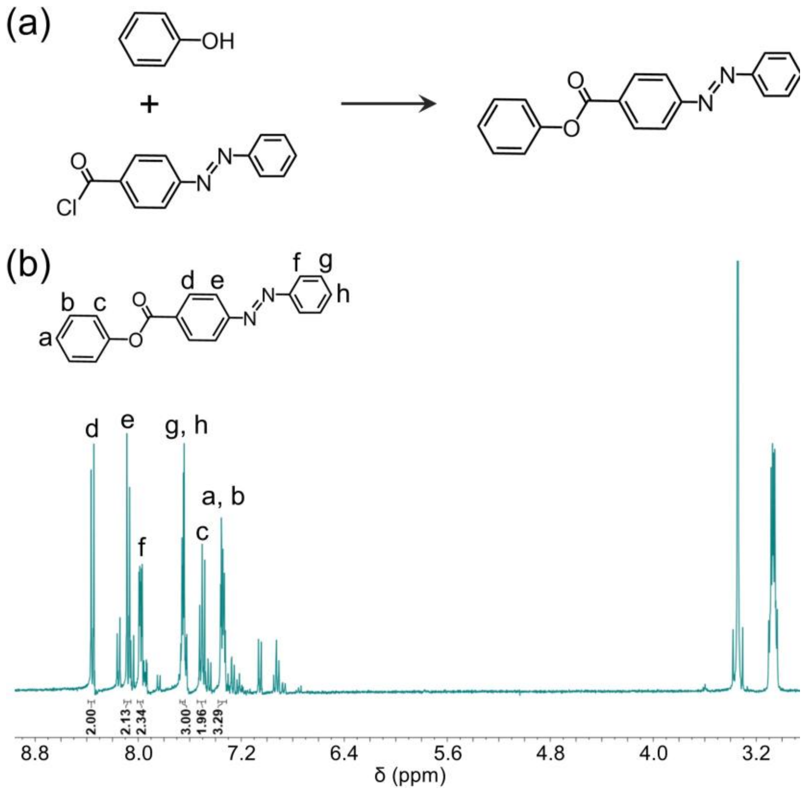

Fig. S5. (a) Synthetic route and (b) ^{1}H NMR spectrum of model compound.

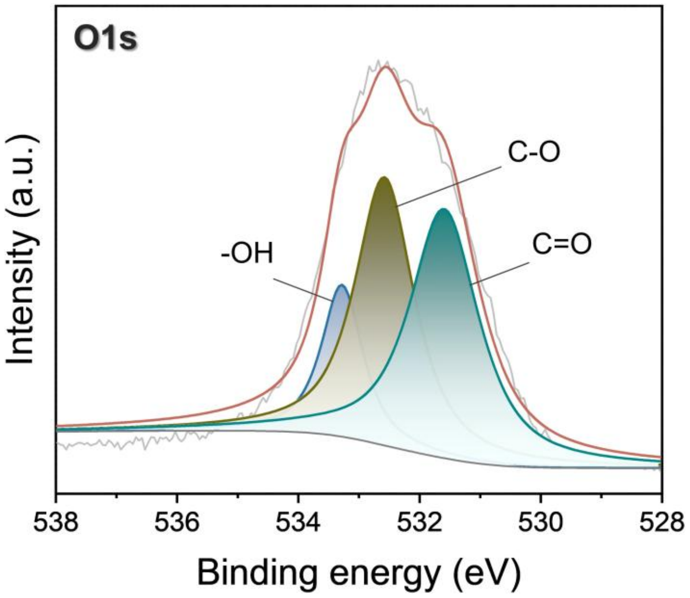

Fig. S6. O 1s spectrum of the 3D-Azo-COF powder.

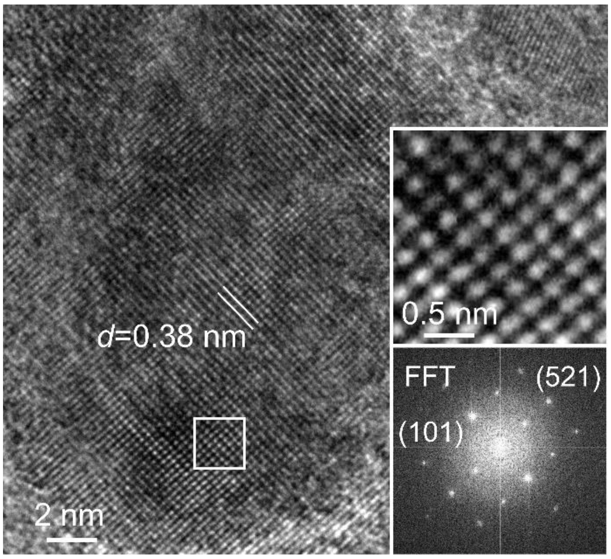

Fig. S7. High-resolution TEM image of 3D-Azo-COF. Marked area is cropped and scaled up in the inset, and the FFT of the cropped area is indexed as the [001] zone axis.

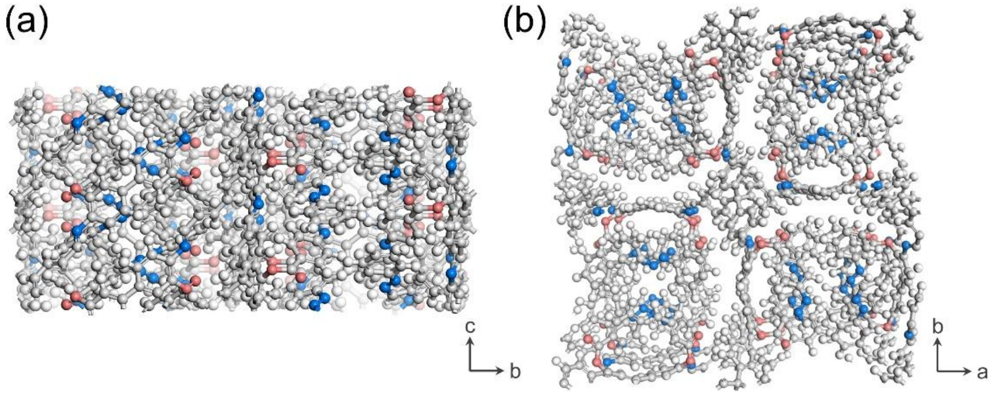

Fig. S8. Crystal structures of 3D-Azo-COF along (a) *a* and (b) *c* axis.

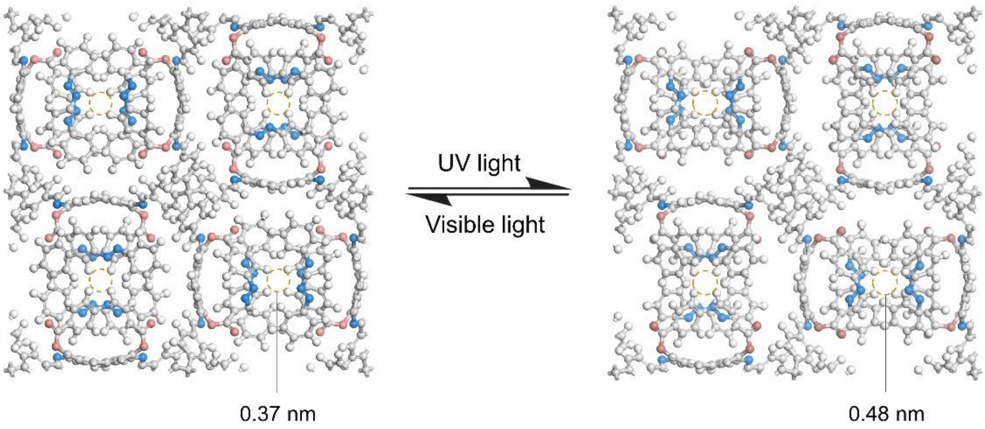

Fig. S9. Simulated structural change of 3D-Azo-COF upon exposure to UV and visible light.

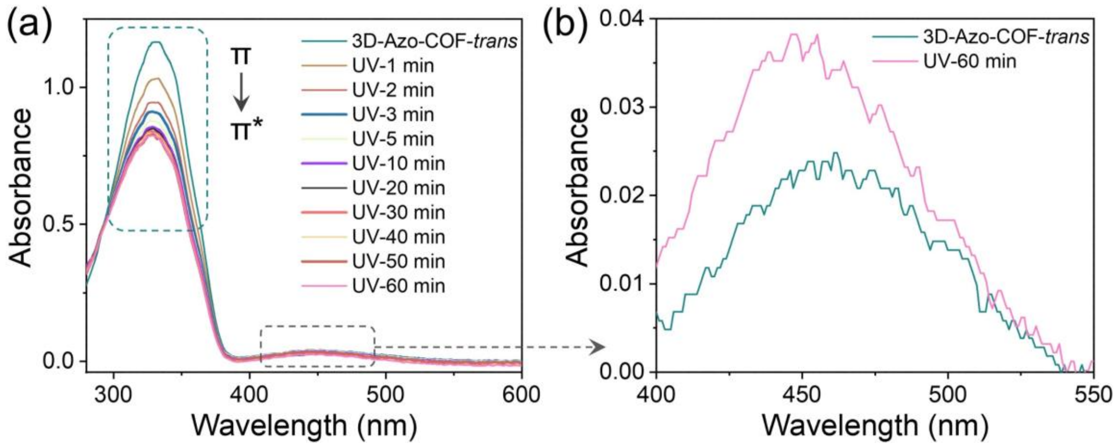

Fig. S10. (a) UV-Vis spectral changes of *trans*-to-*cis* isomerization of 3D-Azo-COF-*trans* upon UV light exposure at 320 nm. (b) Magnified UV-Vis spectra suggesting an intensity change at 452 nm.

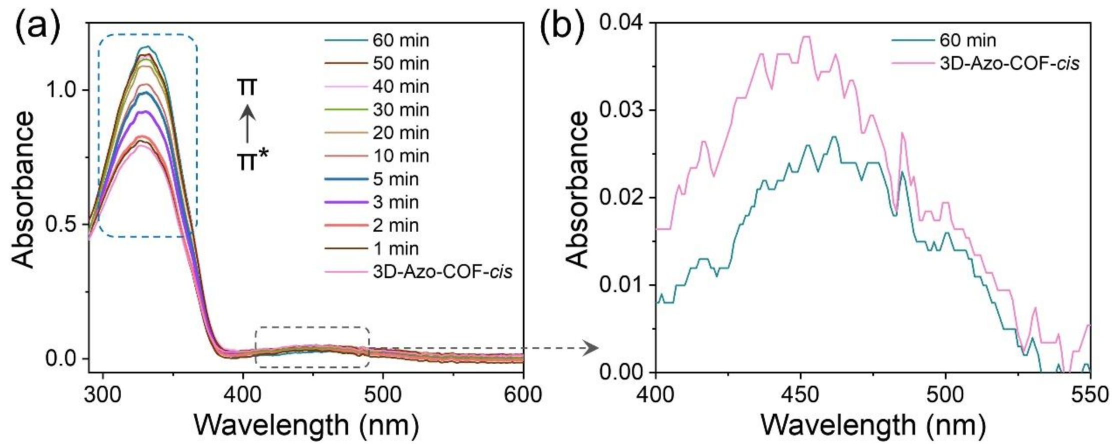

Fig. S11. (a) UV-Vis spectral changes of *cis*-to-*trans* isomerization of 3D-Azo-COF upon visible light exposure at 320 nm. (b) Magnified UV-Vis spectra suggesting an intensity change at 450 nm.

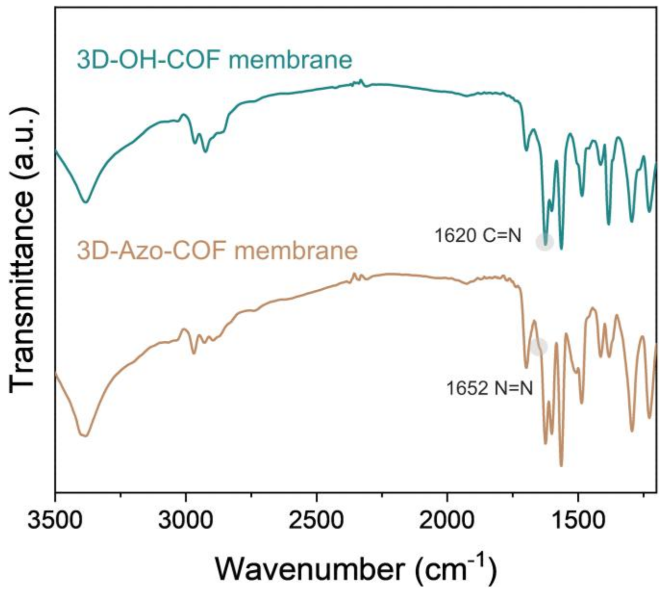

Fig. S12. FTIR spectra of 3D-OH-COF and 3D-Azo-COF membranes.

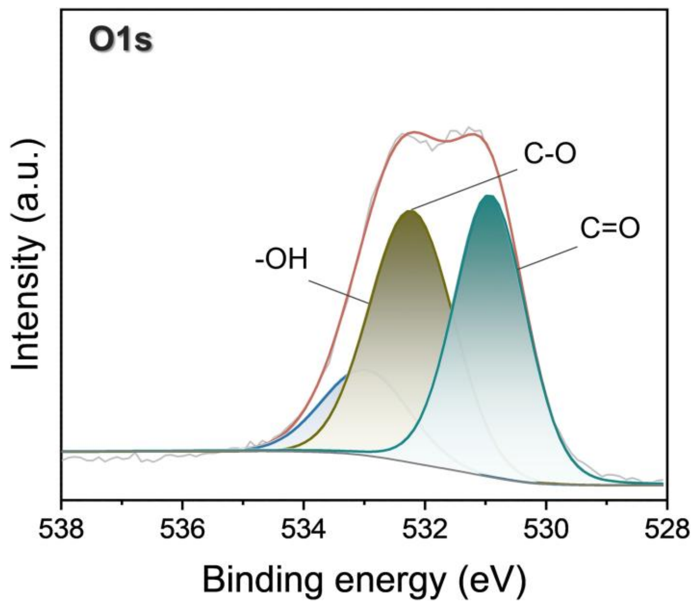

Fig. S13. O 1s spectrum of the 3D-Azo-COF membrane.

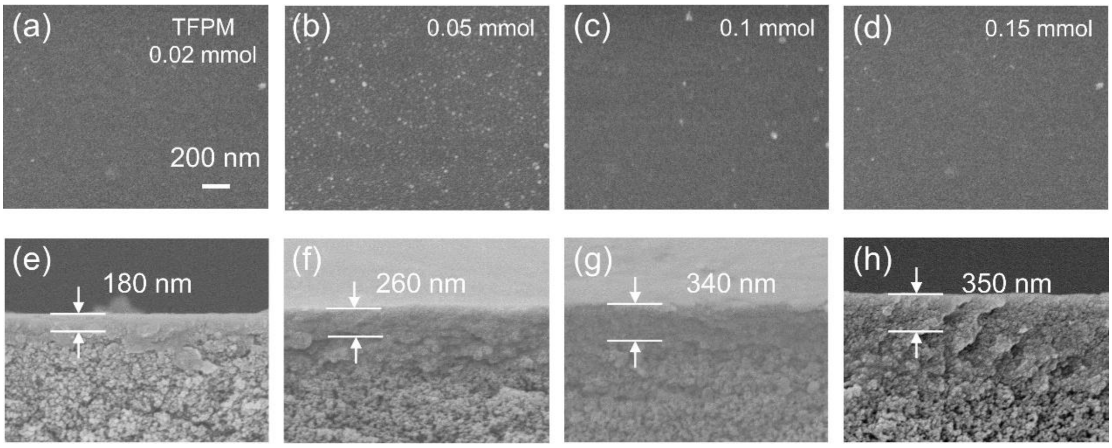

Fig. S14. Surface and cross-sectional SEM images of the 3D-Azo-COF membranes synthesized with different TFPM concentrations: (a, e) 0.02 mmol, (b, f) 0.05 mmol, (c, g) 0.1 mmol, (d, h) 0.15 mmol. The scale in (a) applies to all images.

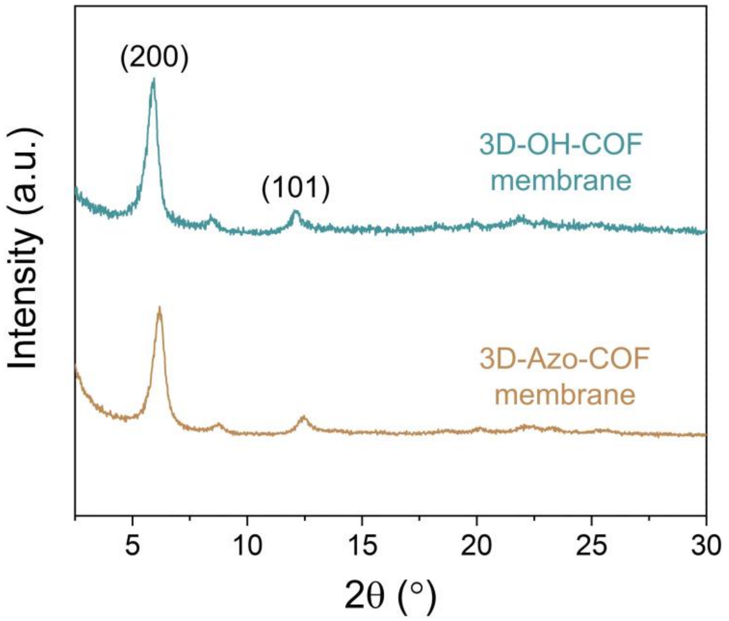

Fig. S15. GIXRD patterns of the membranes.

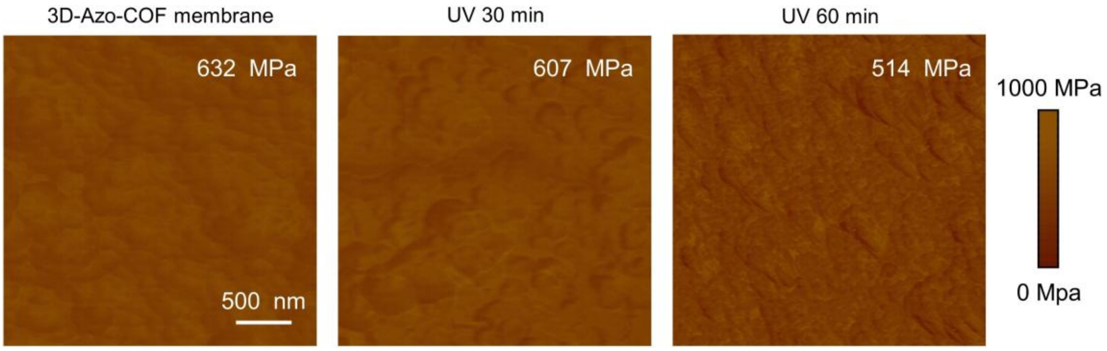

Fig. S16. Young's moduli of the 3D-Azo-COF membrane upon exposure to the UV-light. The scale bar applies to all images.

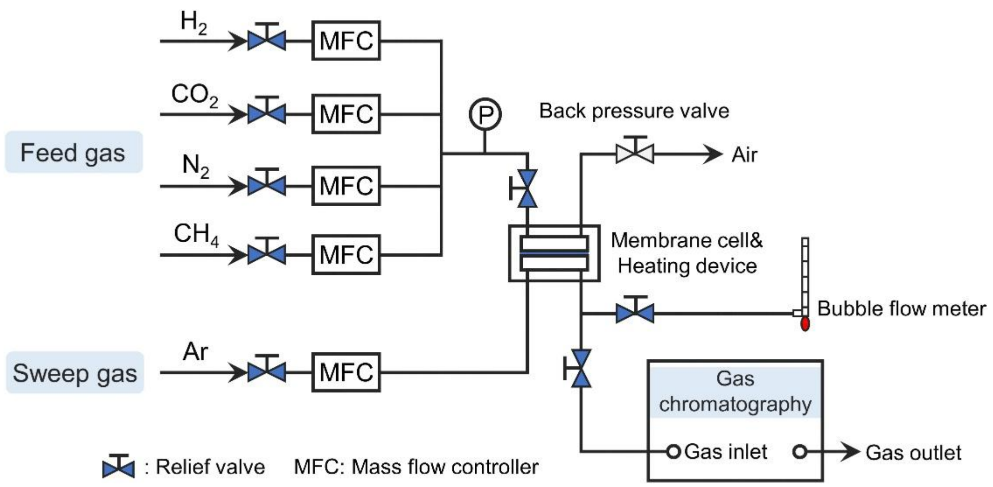

Fig. S17. Schematic diagram of the permeation equipment for single gas and binary gas permeation tests.

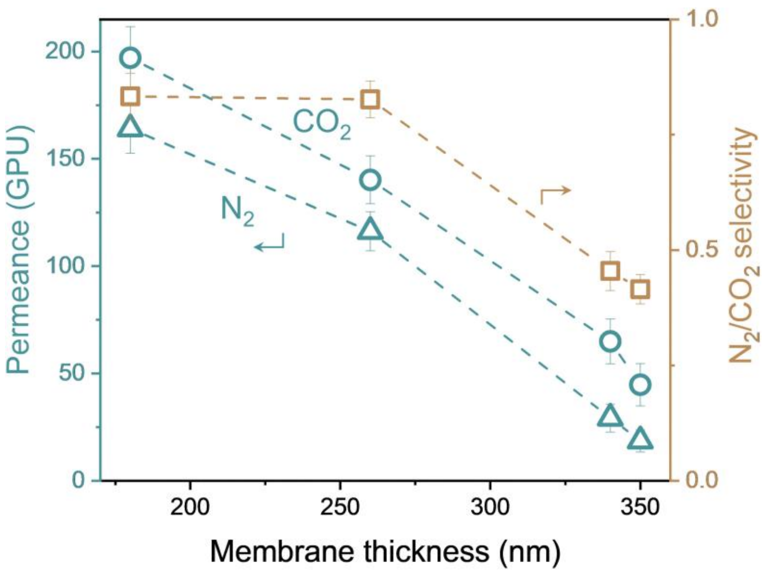

Fig. S18. Effect of membrane thickness on the N_2/CO_2 separation performance of 3D-Azo-COF membranes.

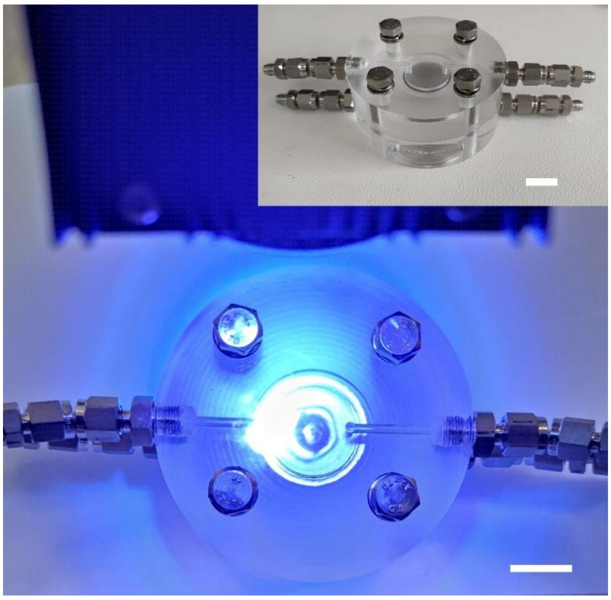

Fig. S19. Digital photos of the device for gas permeation tests. The inset shows the static gas permeation without external lights. Scale bar = 2 cm.

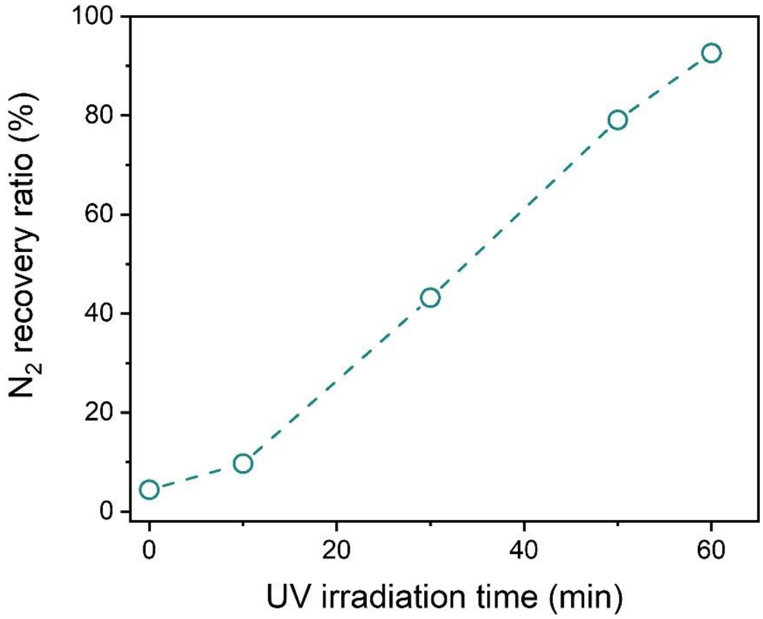

Fig. S20. The effect of UV-light irradiation time on the N_2 recovery ratio.

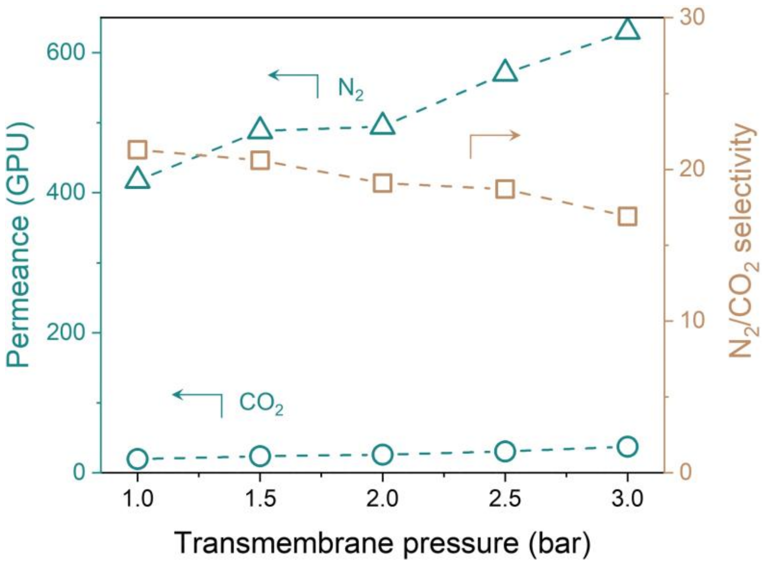

Fig. S21. The change of N_2/CO_2 separation performance of the 3D-Azo-COF-*cis* membrane at varied transmembrane pressure.

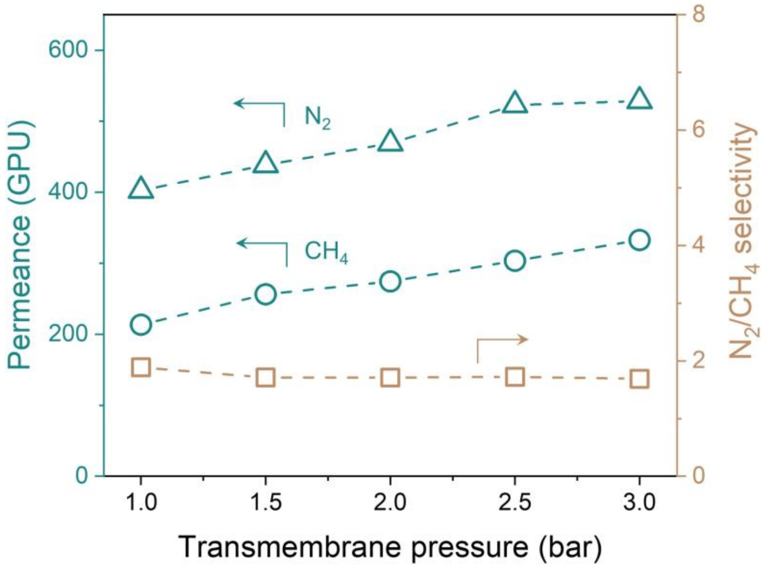

Fig. S22. The change of N_2/CH_4 separation performance of the 3D-Azo- COF-*cis* membrane at varied transmembrane pressures.

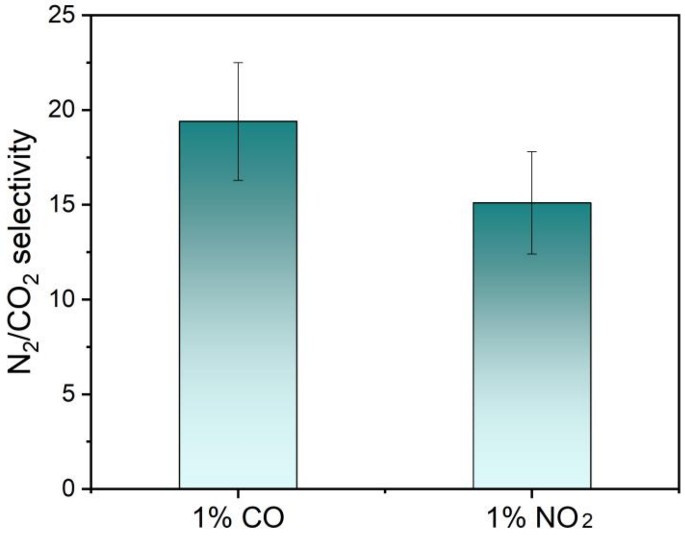

Fig. S23. Effect of purity of feed gases on the N_2/CO_2 selectivity.

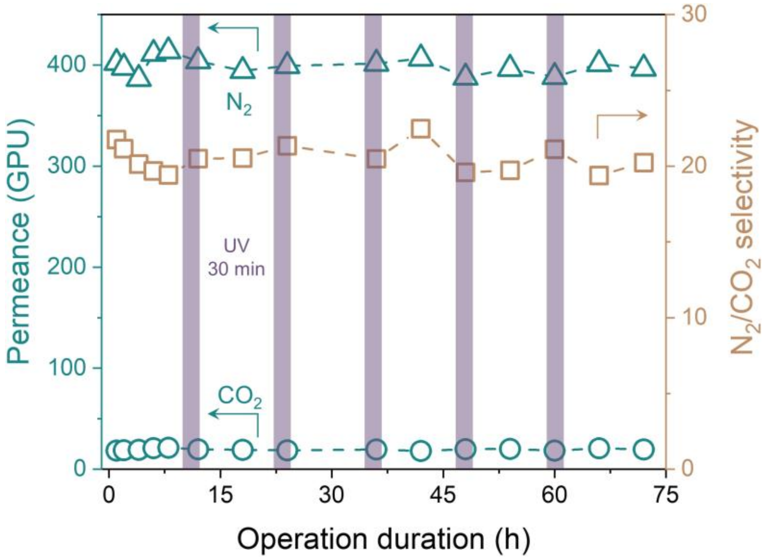

Fig. S24. Performance in long-term separation under scheduled UV-light activation every 6 h.

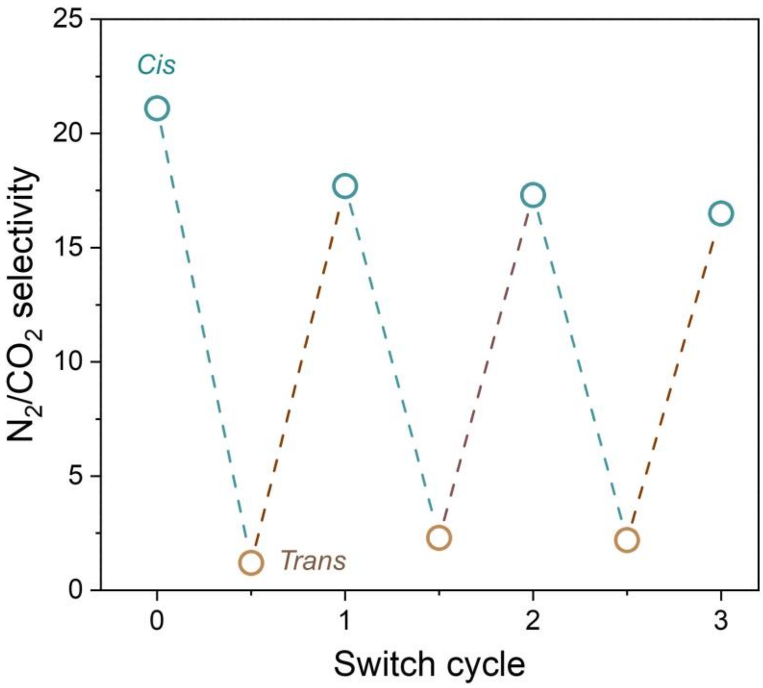

Fig. S25. Switchable N_2/CO_2 separation under cyclic exposure with 365 nm and 450 nm lights for 1 h.

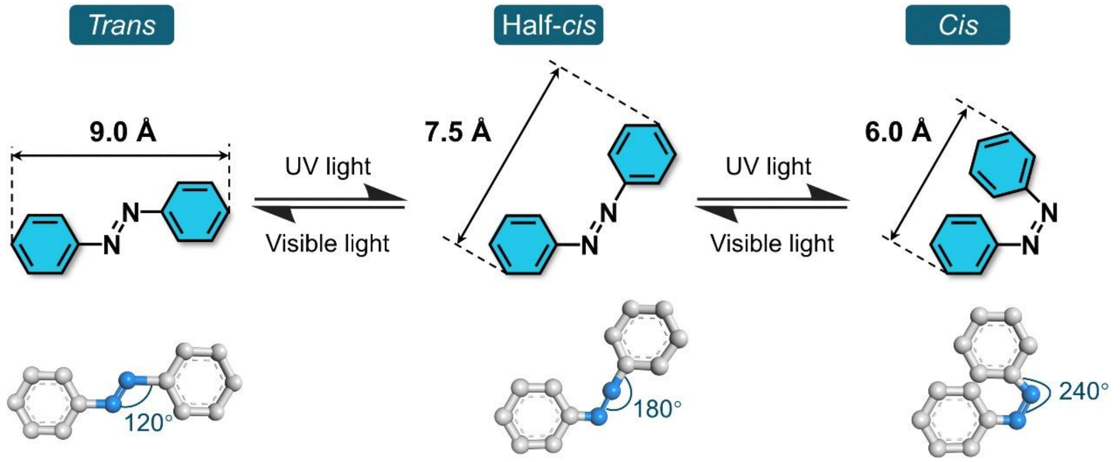

Fig. S26. Structures of the azobenzene with isomerization paths from *trans* to *cis* states. The simulated structures show the variation of bond angle under light exposure.

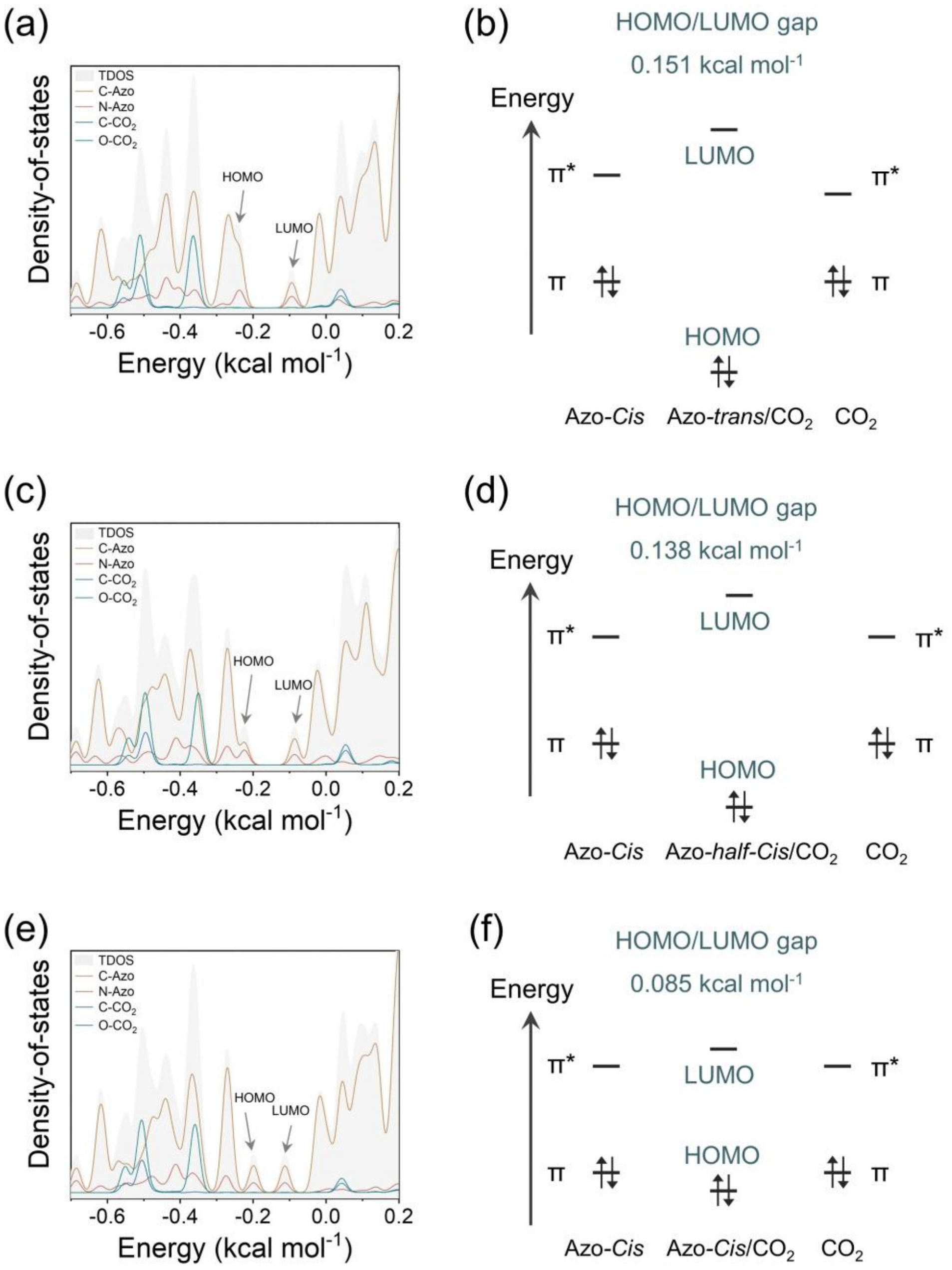

Fig. S27. The total (gray) and atom partial (yellow, red, cyan, and blue) DOS of (a) Azo-*trans*/CO_2, (c) Azo-half-*cis*/CO_2 and (e) Azo-*cis*/CO_2 complexes. Schematic atom interaction diagrams of (b) Azo-*trans*/CO_2, (d) Azo-half-*cis*/CO_2 and (f) Azo-*cis*/CO_2 complexes.

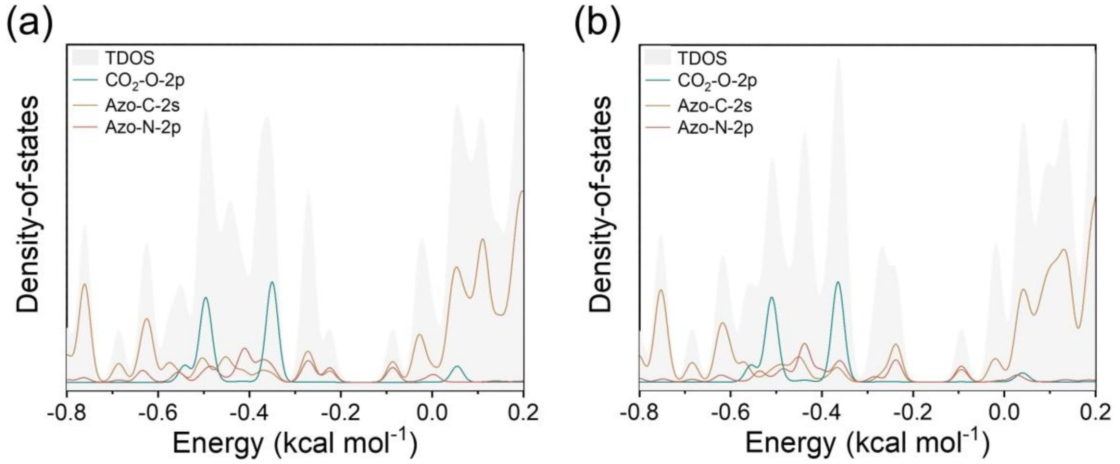

Fig. S28. The total (gray) and orbital partial (yellow, red, cyan, and blue) DOS of (a) Azo-*trans*/CO_2 and (b) Azo-*cis*/CO_2 complexes.

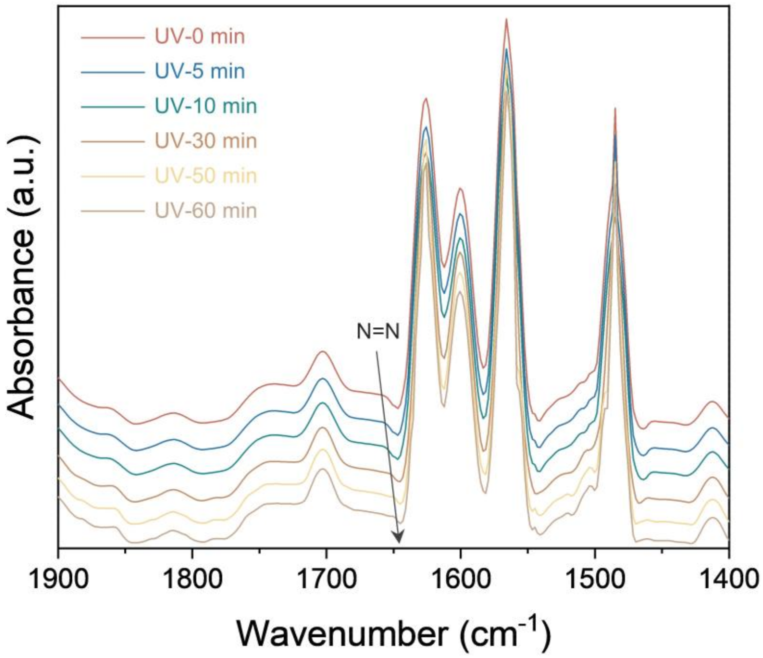

Fig. S29. In situ FTIR spectra recorded for 3D-Azo-COF at CO_2 atmosphere upon different UV-light irradiation time.

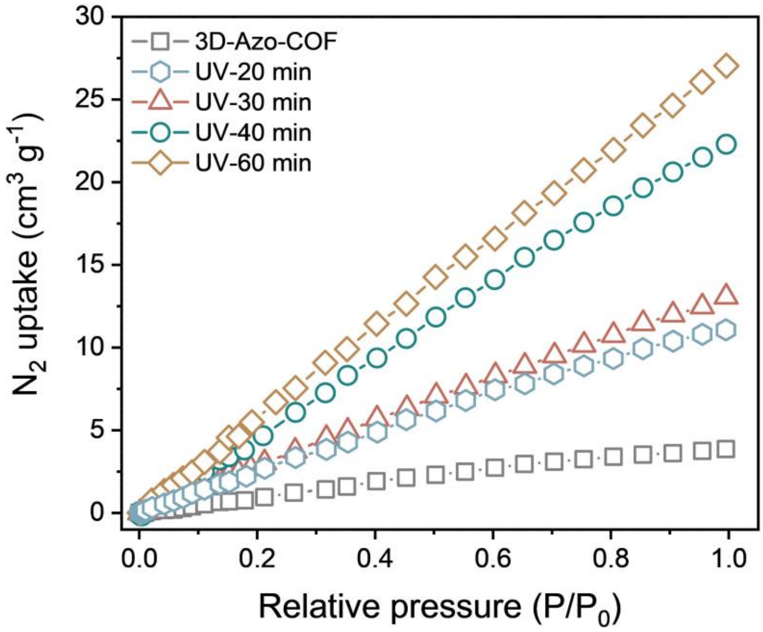

Fig. S30. N_2 uptake isotherms at 298 K for 3D-Azo-COF powders upon different UV-light irradiation time.

Table S1 Physical property of gases(*40*).

Gas	Polarizability (Å^3)	Kinetic diameter (Å)
H_2	0.787	2.89
N_2	1.710	3.64
CO	1.872	3.72
CH_4	2.448	3.80
CO_2	2.911	3.30
SO_2	3.218	4.12

Table S2 Summary of the membrane performance comparison for N_2/CO_2 separation.

Membrane types	Membrane Materials	N_2 permeance (GPU)	N_2/CO_2 selectivity	Ref.
Porous materials	PVA/ZIF-L	78	28	(*41*)
	C_3N_4	50	1.8	(*42*)
	TpTG/TpPa-SO_3/COF-LZU1	64	0.8	(*43*)
	HKUST-1	418.7	0.9	(*44*)
	COF-LZU1	177.1	0.3	(*45*)
	ZIF-8	63	12.9	(*46*)
	2D ZIF-L	71.6	3.0	(*47*)
	SAPO-34/alumina	94	0.2	(*48*)
	PDMS/MOF	450	5.7	(*49*)
	PEI/MCM-48	27.3	3.0	(*50*)
Conventional polymers	Na-X-ACB	0.003	32	(*51*)
	PVP/PAN	8.8	17.8	(*52*)
	PVP/CuO/BQ	13.3	23.1	(*53*)
	rGO-La-2-1	90.2	11.4	(*54*)
	MXene	328	4.7	(*55*)
	2D graphene	60.3	2.9	(*56*)
Photo-responsive membrane	3D-Azo-COF	442.6	27.4	This work

REFERENCES AND NOTES

1. C. G. Gruber, L. Frey, R. Guntermann, D. D. Medina, E. Cortés, Early stages of covalent organic framework formation imaged in operando. *Nature* **630**, 872–877 (2024).

2. M. Feng, C. Xing, Y. Jin, X. Feng, Y. Zhang, B. Wang, Reticular chemistry for enhancing bioentity stability and functional performance. *J. Am. Chem. Soc.* **146**, 32883–32905 (2024).

3. J. Han, J. Feng, J. Kang, J.-M. Chen, X.-Y. Du, S.-Y. Ding, L. Liang, W. Wang, Fast growth of single-crystal covalent organic frameworks for laboratory x-ray diffraction. *Science* **383**, 1014–1019 (2024).

4. S. Li, S. Xu, E. Lin, T. Wang, H. Yang, J. Han, Y. Zhao, Q. Xue, P. Samorì, Z. Zhang, T. Zhang, Synthesis of single-crystalline sp^2-carbon-linked covalent organic frameworks through imine-to-olefin transformation. *Nat. Chem.* **17**, 226–232 (2025).

5. W. Zhang, L. Chen, S. Dai, C. Zhao, C. Ma, L. Wei, M. Zhu, S. Y. Chong, H. Yang, L. Liu, Y. Bai, M. Yu, Y. Xu, X.-W. Zhu, Q. Zhu, S. An, R. S. Sprick, M. A. Little, X. Wu, S. Jiang, Y. Wu, Y.-B. Zhang, H. Tian, W.-H. Zhu, A. I. Cooper, Reconstructed covalent organic frameworks. *Nature* **604**, 72–79 (2022).

6. X. Pang, B. Shi, Y. Liu, Y. Li, Y. Zhang, T. Wang, S. Xu, X. Wang, Z. Liu, N. Xing, X. Liang, Z. Zhu, C. Fan, Y. Liu, H. Wu, Z. Jiang, Phosphorylated covalent organic framework membranes toward ultrafast single lithium-ion transport. *Adv. Mater.* **36**, e2413022 (2024).

7. X. Tian, L. Cao, K. Zhang, R. Zhang, X. Li, C. Yin, S. Wang, Molecular weaving towards flexible covalent organic framework membranes for efficient gas separations. *Angew. Chem. Int. Ed.* **64**, e202416864 (2025).

8. A. Knebel, J. Caro, Metal-organic frameworks and covalent organic frameworks as disruptive membrane materials for energy-efficient gas separation. *Nat. Nanotechnol.* **17**, 911–923 (2022).

9. H. Yang, H. Zhang, C. Kang, C. Ji, D. Shi, D. Zhao, Solvent-responsive covalent organic framework membranes for precise and tunable molecular sieving. *Sci. Adv.* **10**, eads0260 (2024).

10. H. Fan, H. Wang, M. Peng, H. Meng, A. Mundstock, A. Knebel, J. Caro, Pore-in-pore engineering in a covalent organic framework membrane for gas separation. *ACS Nano* **17**, 7584–7594 (2023).

11. F. Yang, J. Guo, C. Han, J. Huang, Z. Zhou, S.-P. Sun, Y. Zhang, L. Shao, Turing covalent organic framework membranes via heterogeneous nucleation synthesis for organic solvent nanofiltration. *Sci. Adv.* **10**, eadr9260 (2024).

12. Y. Hu, B. Sengupta, H. Long, L. J. Wayment, R. Ciora, Y. Jin, J. Wu, Z. Lei, K. Friedman, H. Chen, M. Yu, W. Zhang, Molecular recognition with resolution below 0.2 angstroms through thermoregulatory oscillations in covalent organic frameworks. *Science* **384**, 1441–1447 (2024).

13. Y. Zhao, X. Tao, J. Lin, S. Lin, Azobenzene functionalized organic covalent frameworks: Controlled morphologies and photo-regulated adsorption. *Adv. Funct. Mater.* **33**, 2302225 (2023).

14. F. Auras, L. Ascherl, V. Bon, S. M. Vornholt, S. Krause, M. Döblinger, D. Bessinger, S. Reuter, K. W. Chapman, S. Kaskel, R. H. Friend, T. Bein, Dynamic two-dimensional covalent organic frameworks. *Nat. Chem.* **16**, 1373–1380 (2024).

15. C. Yin, L. Liu, Z. Zhang, Y. Du, Y. Wang, Photo-induced geometry and polarity gradients in covalent organic frameworks enabling fast and durable molecular separations. *Small* **20**, 2309329 (2024).

16. Y. Li, B. Xue, J. Yang, J. Jiang, J. Liu, Y. Zhou, J. Zhang, M. Wu, Y. Yuan, Z. Zhu, Z. J. Wang, Y. Chen, Y. Harabuchi, T. Nakajima, W. Wang, S. Maeda, J. P. Gong, Y. Cao, Azobenzene as a photoswitchable mechanophore. *Nat. Chem.* **16**, 446–455 (2023).

17. F. A. Jerca, V. V. Jerca, R. Hoogenboom, Advances and opportunities in the exciting world of azobenzenes. *Nat. Rev. Chem.* **6**, 51–69 (2022).

18. J. Liu, S. Wang, T. Huang, P. Manchanda, E. Abou-Hamad, S. P. Nunes, Smart covalent organic networks (CONs) with "on-off-on" light-switchable pores for molecular separation. *Sci. Adv.* **6**, eabb3188 (2020).

19. C. Yin, Z. Zhang, Z. Si, X. Shi, Y. Wang, Smart covalent organic frameworks with intrapore azobenzene groups for light-gated ion transport. *Chem. Mater.* **34**, 9212–9220 (2022).

20. G. Das, T. Prakasam, M. A. Addicoat, S. K. Sharma, F. Ravaux, R. Mathew, M. Baias, R. Jagannathan, M. A. Olson, A. Trabolsi, Azobenzene-equipped covalent organic framework: Light-operated reservoir. *J. Am. Chem. Soc.* **141**, 19078–19087 (2019).

21. G. Wang, Y. Feng, X. Ye, Z. Li, S. Tao, D. Jiang, Light-gating crystalline porous covalent organic frameworks. *J. Am. Chem. Soc.* **146**, 10953–10962 (2024).

22. D. Urban, N. Marcucci, C. H. Wölfle, J. Torgersen, D. R. Hjelme, E. Descrovi, Polarization-driven reversible actuation in a photo-responsive polymer composite. *Nat. Commun.* **14**, 6843 (2023).

23. Q. Huang, Z. Zhan, R. Sun, J. Mu, B. Tan, C. Wu, Light triggered pore size tuning in photoswitching covalent triazine frameworks for low energy CO_2 capture. *Angew. Chem. Int. Ed.* **62**, e202305500 (2023).

24. Y. Feng, G. Wang, R. Liu, X. Ye, S. Tao, M. A. Addicoat, Z. Li, Q. Jiang, D. Jiang, Photoresponsive covalent organic frameworks: Visible-light controlled conversion of porous structures and its impacts. *Angew. Chem. Int. Ed.* **63**, e202400009 (2024).

25. Y. Yin, Y. Zhang, X. Zhou, B. Gui, W. Wang, W. Jiang, Y.-B. Zhang, J. Sun, C. Wang, Ultrahigh–surface area covalent organic frameworks for methane adsorption. *Science* **386**, 693–696 (2024).

26. F. Jin, E. Lin, T. Wang, S. Geng, L. Hao, Q. Zhu, Z. Wang, Y. Chen, P. Cheng, Z. Zhang, Rationally fabricating three-dimensional covalent organic frameworks for propyne/propylene separation. *J. Am. Chem. Soc.* **144**, 23081–23088 (2022).

27. F. Chen, H. Zheng, Y. Yusran, H. Li, S. Qiu, Q. Fang, Exploring high-connectivity three-dimensional covalent organic frameworks: Topologies, structures, and emerging applications. *Chem. Soc. Rev.* **54**, 484–514 (2025).

28. Y. Xie, W. Wang, Z. Zhang, J. Li, B. Gui, J. Sun, D. Yuan, C. Wang, Fine-tuning the pore environment of ultramicroporous three-dimensional covalent organic frameworks for efficient one-step ethylene purification. *Nat. Commun.* **15**, 3008 (2024).

29. X. Guan, F. Chen, S. Qiu, Q. Fang, Three-dimensional covalent organic frameworks: From synthesis to applications. *Angew. Chem. Int. Ed.* **62**, e202213203 (2022).

30. Q. Lu, Y. Ma, H. Li, X. Guan, Y. Yusran, M. Xue, Q. Fang, Y. Yan, S. Qiu, V. Valtchev, Postsynthetic functionalization of three-dimensional covalent organic frameworks for selective extraction of lanthanide ions. *Angew. Chem. Int. Ed.* **57**, 6042–6048 (2018).

31. X. Shi, Z. Zhang, S. Fang, J. Wang, Y. Zhang, Y. Wang, Flexible and robust three-dimensional covalent organic framework membranes for precise separations under extreme conditions. *Nano Lett.* **21**, 8355–8362 (2021).

32. S. E. Neumann, J. Kwon, C. Gropp, L. Ma, R. Giovine, T. Ma, N. Hanikel, K. Wang, T. Chen, S. Jagani, R. O. Ritchie, T. Xu, O. M. Yaghi, The propensity for covalent organic frameworks to template polymer entanglement. *Science* **383**, 1337–1343 (2024).

33. R. M. Zhu, Y. Liu, W. K. Han, J. D. Feng, J. Zhang, H. Pang, J. Zhang, Z. G. Gu, Three-dimensional covalent organic frameworks based on linear and trigonal linkers for high-performance H_2O_2 photosynthesis. *Angew. Chem. Int. Ed.* **64**, e202412890 (2024).

34. J. Gemen, J. R. Church, T.-P. Ruoko, N. Durandin, M. J. Białek, M. Weißenfels, M. Feller, M. Kazes, M. Odaybat, V. A. Borin, R. Kalepu, Y. Diskin-Posner, D. Oron, M. J. Fuchter, A.

Priimagi, I. Schapiro, R. Klajn, Disequilibrating azobenzenes by visible-light sensitization under confinement. *Science* **381**, 1357–1363 (2023).

35. L. Cheng, Y. Guo, Q. Liu, G. Liu, R. Li, X. Chen, H. Zeng, G. Liu, W. Jin, Metal confined in 2D membranes for molecular recognition and sieving towards ethylene/ethane separation. *Adv. Mater.* **34**, 2206349 (2022).

36. R. Yang, Y. Wang, J.-W. Cao, Z.-M. Ye, T. Pham, K. A. Forrest, R. Krishna, H. Chen, L. Li, B.-K. Ling, T. Zhang, T. Gao, X. Jiang, X.-O. Xu, Q.-H. Ye, K.-J. Chen, Hydrogen bond unlocking-driven pore structure control for shifting multi-component gas separation function. *Nat. Commun.* **15**, 804 (2024).

37. J. K. Yu, C. Bannwarth, R. Liang, E. G. Hohenstein, T. J. Martinez, Nonadiabatic dynamics simulation of the wavelength-dependent photochemistry of azobenzene excited to the nπ* and ππ* excited states. *J. Am. Chem. Soc.* **142**, 20680–20690 (2020).

38. Q. Xu, J. Han, F. Tian, X. Zhao, J. Rong, J. Zhang, P. She, J.-S. Qin, H. Rao, Synergistic bifunctional covalent organic framework for efficient photocatalytic CO_2 reduction and water oxidation. *J. Am. Chem. Soc.* **147**, 10587–10597 (2025).

39. I. C. D. Merritt, D. Jacquemin, M. Vacher, *cis* → *trans* photoisomerisation of azobenzene: A fresh theoretical look. *Phys. Chem. Chem. Phys.* **23**, 19155–19165 (2021).

40. T. Ashirov, J. S. Siena, M. Zhang, A. Ozgur Yazaydin, M. Antonietti, A. Coskun, Fast light-switchable polymeric carbon nitride membranes for tunable gas separation. *Nat. Commun.* **13**, 7299 (2022).

41. H. Li, L. Han, J. Hou, J. Liu, Y. Zhang, Oriented zeolitic imidazolate framework membranes within polymeric matrices for effective N_2/CO_2 separation. *J. Membr. Sci.* **572**, 82–91 (2019).

42. Y. Zhou, Y. Wu, H. Wu, J. Xue, L. Ding, R. Wang, H. Wang, Fast hydrogen purification through graphitic carbon nitride nanosheet membranes. *Nat. Commun.* **13**, 5852 (2022).

43. Y. Ying, S. B. Peh, H. Yang, Z. Yang, D. Zhao, Ultrathin covalent organic framework membranes via a multi-interfacial engineering strategy for gas separation. *Adv. Mater.* **34**, e2104946 (2022).

44. H. Guo, G. Zhu, I. J. Hewitt, S. Qiu, "Twin Copper Source" growth of metal−organic framework membrane: $Cu_3(BTC)_2$ with high permeability and selectivity for recycling H_2. *J. Am. Chem. Soc.* **131**, 1646–1647 (2009).

45. H. Fan, A. Mundstock, A. Feldhoff, A. Knebel, J. Gu, H. Meng, J. Caro, Covalent organic framework-covalent organic framework bilayer membranes for highly selective gas separation. *J. Am. Chem. Soc.* **140**, 10094–10098 (2018).

46. G. Xu, J. Yao, K. Wang, L. He, P. A. Webley, C.-s. Chen, H. Wang, Preparation of ZIF-8 membranes supported on ceramic hollow fibers from a concentrated synthesis gel. *J. Membr. Sci.* **385-386**, 187–193 (2011).

47. Z. Zhong, J. Yao, R. Chen, Z. Low, M. He, J. Z. Liu, H. Wang, Oriented two-dimensional zeolitic imidazolate framework-L membranes and their gas permeation properties. *J. Mater. Chem. A* **3**, 15715–15722 (2015).

48. K. Kgaphola, I. Sigalas, M. O. Daramola, Synthesis and characterization of nanocomposite SAPO-34/ceramic membrane for post-combustion CO_2 capture. *Asia Pac. J. Chem. Eng.* **12**, 894–904 (2017).

49. W. Li, G. Zhang, C. Zhang, Q. Meng, Z. Fan, C. Gao, Synthesis of trinity metal–organic framework membranes for CO_2 capture. *Chem. Commun.* **50**, 3214–3216 (2014).

50. P. Kumar, S. Kim, J. Ida, V. V. Guliants, Polyethyleneimine-modified MCM-48 membranes: Effect of water vapor and feed concentration on N_2/CO_2 selectivity. *Ind. Eng. Chem. Res.* **47**, 201–208 (2008).

51. K. Weh, M. Noack, K. Hoffmann, K. P. Schröder, J. Caro, Change of gas permeation by photoinduced switching of zeolite-azobenzene membranes of type MFI and FAU. *Micropor. Mesopor. Mater.* **54**, 15–26 (2002).

52. T. G. Kwon, J. Lee, O. H. Jo, B. G. Kang, S. W. Kang, Charged surface of polyacrylonitrile colloid and its application to N_2/CO_2 separation. *Macromol. Chem. Phys.* **225**, 2300323 (2023).

53. J. Lee, H. Sohn, S. W. Kang, Surface of CuO nanoparticles modified by p-benzoquinone for N_2-selective membrane. *Membranes* **12**, 1229 (2022).

54. S. Yu, B. Qin, F. Yang, M. Xie, L. Xue, Z. Zhao, K. Wang, Unlocking the limits of diffusion and adsorption of metal-crosslinked reduced graphene oxide membranes for gas separation. *Appl. Surf. Sci.* **586**, 152868 (2022).

55. J. Shen, G. Liu, Y. Ji, Q. Liu, L. Cheng, K. Guan, M. Zhang, G. Liu, J. Xiong, J. Yang, W. Jin, 2D mxene nanofilms with tunable gas transport channels. *Adv. Funct. Mater.* **28**, 1801511 (2018).

56. F. A. Nezhad, N. Han, Z. Shen, Y. Jin, Y. Wang, N. Yang, S. Liu, Experimental and theoretical exploration of gas permeation mechanism through 2D graphene (not graphene oxides) membranes. *J. Membr. Sci.* **601**, 117883 (2020).
